# Parkinson’s disease-implicated kinases in the brain; insights into disease pathogenesis

**DOI:** 10.3389/fnmol.2014.00057

**Published:** 2014-06-24

**Authors:** Nicolas Dzamko, Jinxia Zhou, Yue Huang, Glenda M. Halliday

**Affiliations:** ^1^School of Medical Sciences, University of New South WalesKensington, NSW, Australia; ^2^Neuroscience Research AustraliaRandwick, NSW, Australia

**Keywords:** kinase, LRRK2, JNK, PLK, PINK1, GAK, MAPK, brain

## Abstract

Substantial evidence implicates abnormal protein kinase function in various aspects of Parkinson’s disease (PD) etiology. Elevated phosphorylation of the PD-defining pathological protein, α-synuclein, correlates with its aggregation and toxic accumulation in neurons, whilst genetic missense mutations in the kinases PTEN-induced putative kinase 1 and leucine-rich repeat kinase 2, increase susceptibility to PD. Experimental evidence also links kinases of the phosphoinositide 3-kinase and mitogen-activated protein kinase signaling pathways, amongst others, to PD. Understanding how the levels or activities of these enzymes or their substrates change in brain tissue in relation to pathological states can provide insight into disease pathogenesis. Moreover, understanding when and where kinase dysfunction occurs is important as modulation of some of these signaling pathways can potentially lead to PD therapeutics. This review will summarize what is currently known in regard to the expression of these PD-implicated kinases in pathological human postmortem brain tissue.

## INTRODUCTION

Studies of the postmortem human brain have been invaluable in gaining insights into the etiology of Parkinson’s disease (PD), an increasingly common movement disorder resulting from the early selective loss of dopamine producing neurons in the substantia nigra. Dr. Fritz Lewy discovered the eponymous intracellular inclusion bodies synonymous with PD (Rodrigues et al., 2010). Drs. Tretiakoff, Hassler, and others were able to demonstrate degeneration of the substantia nigra in PD (Hassler, 1938). Postmortem brain studies were integral to the experiments of Carlsson and colleagues and their discoveries on the therapeutic potential of exogenous dopamine treatment for PD patients (Carlsson, 1959) and their discoveries on the therapeutic potential of exogenous dopamine treatment for PD patients and more recently, the work of Braak and others has suggested that PD spreads through the brain in a predictable or staged fashion (Braak et al., 2003; Halliday et al., 2008).

The Braak staging hypothesis is modeled on the toxic spread and accumulation of α-synuclein, a 17–18 kDa presynaptic protein encoded by the *SNCA* gene. Point mutations in, or multiplications of, the *SNCA* gene cause familial PD in an autosomal-dominant fashion (Polymeropoulos et al., 1997), whilst genome-wide association studies conclude that common variations in the *SNCA* gene increase the risk of sporadic PD (Pihlstrom and Toft, 2011). Moreover, α-synuclein is the predominant component of Lewy bodies, where it accumulates in an aggregated form (Spillantini et al., 1997). Hence, α-synuclein is proposed as a key protein in the pathogenesis of PD. Accumulating evidence suggests that α-synuclein acts in a prion-like manner, inducing the aggregation of healthy α-synuclein and propagating the spread of PD from neuron to neuron (Olanow and Brundin, 2013). The aggregated and proposed toxic form of α-synuclein is hyperphosphorylated (Oueslati et al., 2010). In disease free conditions only 4% of total α-synuclein is phosphorylated in brain, but in PD and related synucleinopathies, >90% of α-synuclein deposited in Lewy bodies is phosphorylated (Fujiwara et al., 2002; Anderson et al., 2006). In particular, phosphorylation of pathological α-synuclein on serine 129 (S129) is prevalent in PD postmortem brain (Fujiwara et al., 2002; Anderson et al., 2006; Zhou et al., 2011; Lue et al., 2012; Walker et al., 2013). Although the biological consequences of α-synuclein phosphorylation remain inconclusive, there is much interest in the identification of the kinases mediating this event. A number of candidate kinases, including members of the polo-like kinase (PLK), casein kinase (CK), and G protein coupled receptor kinase (GRK) families have subsequently been identified.

In addition to the hyperphosphorylation of α-synuclein, kinase dysfunction is also genetically linked to PD. In particular, missense mutations in the leucine-rich repeat kinase 2 (LRRK2) are causal for autosomal-dominant familial PD (Paisan-Ruiz et al., 2004; Zimprich et al., 2004), whilst multiple mutations in the PTEN-induced putative kinase 1 (PINK1) protein are causative for familial PD in a recessive fashion (Valente et al., 2004). Moreover, common polymorphisms identified by genome-wide association in loci encoding cyclin G-associated kinase (*GAK*) and serine/threonine kinase 39 [*STK39*, more commonly known as STE20-related proline alanine-rich kinase (SPAK)], have implicated these kinases as susceptibility enzymes for sporadic PD (Pankratz et al., 2009; Nalls et al., 2011; Sharma et al., 2012).

Finally a myriad of laboratory studies have focused on kinase signaling in PD. Kinases remain attractive targets for the treatment of many human diseases. Kinases of the MAPK and PI3K signaling pathways including extracellular signal related protein kinase (ERK), c-Jun N-terminal kinase (JNK), p38, protein kinase B (PKB), and mammalian target of rapamycin (mTOR) make particularly attractive targets for PD through their ability to coordinate and regulate cell survival, apoptosis, inflammation, and autophagy.

## KINASES MEDIATING α-SYNUCLEIN S129 PHOSPHORYLATION IN PD

**FIGURE 1:**
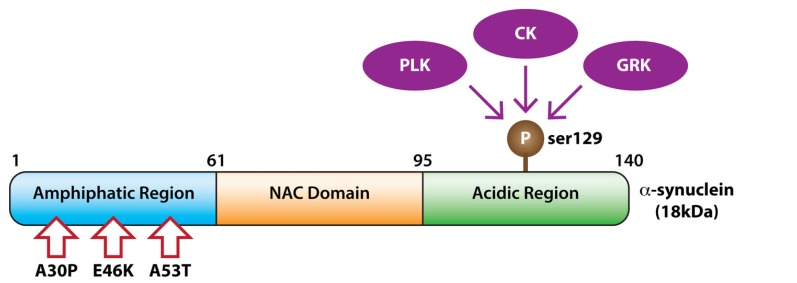
**Kinases phosphorylating α-synuclein**. The domain structure of α-synuclein showing phosphorylation at serine 129 by members of the polo-like kinase (PLK), casein kinase (CK), and G protein coupled receptor kinase (GRK) families. Pathogenic α-synuclein missense mutations are indicated with arrows.

The exact mechanism resulting in the pathological accumulation of S129 phosphorylated α-synuclein is unclear. A number of kinases phosphorylate α-synuclein at this residue *in vitro* with accumulating evidence for a role *in vivo* (**Figure 1**). Understanding how this major pathological protein becomes hyperphosphorylated and the extent to which post-translational modifications impact upon the aggregation and prion-like spread of α-synuclein could provide key insight into PD etiology.

### POLO-LIKE KINASES (PLKs)

Polo-like kinases (PLKs) comprise a serine/threonine kinase family containing an N-terminal kinase catalytic domain and a C-terminal polo-box domain (PBD) that is involved in substrate binding and regulation of kinase activity. Five mammalian PLK family members from three subfamilies have been identified, including the PLK1 subfamily, the PLK4 subfamily, and the PLK2 subfamily (containing PLK2, PLK3, and PLK5; de Carcer et al., 2011b). The study of PLKs has focused primarily on their critical roles in the cell cycle (Winkles and Alberts, 2005); however, recent studies suggest PLKs also have important roles in terminally differentiated cells of the nervous system (Seeburg et al., 2005).

In particular, PLKs 1–3 are capable of phosphorylating α-synuclein (de Carcer et al., 2011a, b). Comparative studies suggest that PLK2 and PLK3 directly phosphorylate α-synuclein at Ser129 *in vitro* with high stoichiometry, whilst PLK4 is unable to phosphorylate α-synuclein at this residue (Anderson et al., 2006; Inglis et al., 2009). The low kinase activity of PLK4 against α-synuclein, and other substrates, is partially explained by its unique structure, with only a single polo-box in the PBD, resulting in a much-reduced electropositive environment in its substrate-binding site (Mbefo et al., 2010). Human PLK5 lacks a functional kinase domain due to a premature stop codon in exon 6 and is therefore unable to phosphorylate α-synuclein.

Increasing PLK2 or PLK3 significantly up-regulates α-synuclein Ser129 phosphorylation (Mbefo et al., 2010; Waxman and Giasson, 2011), whilst their inhibition or reduction remarkably decreases α-synuclein phosphorylation in both cell and animal models (Inglis et al., 2009; Waxman and Giasson, 2011). This has led to efforts to generate small molecule PLK inhibitors for potential therapeutic use (Bowers et al., 2013; Fitzgerald et al., 2013; Bergeron et al., 2014). The utility of such compounds, however, has been questioned by a recent study showing that Ser129 phosphorylation by PLK2 is required for autophagic degradation of α-synuclein (Oueslati et al., 2013). In this study overexpression of PLK2, as opposed to inhibition, prevented the toxic accumulation of α-synuclein in rodent models, suggesting more work is required to delineate the exact role of PLKs in α-synuclein pathology.

In addition, studies investigating the association of PLKs with α-synuclein pathology / phosphorylation in human brain are lacking. The central nervous system has relatively high levels of PLK2, 3, and 5, low levels of PLK1 and seems to lack PLK4 (Winkles and Alberts, 2005; de Carcer et al., 2011a, b). PLK2 and PLK3 are expressed in most regions of the brain, but surprisingly there is almost no expression of either PLK2 or PLK3 in the cerebellum (Winkles and Alberts, 2005; de Carcer et al., 2011b). Whether PLK2 or PLK3, the main family members that can phosphorylate α-synuclein are increased, or indeed more active, in PD brain remains to be determined. The recent identification of autophosphorylation sites on PLK2 (Rozeboom and Pak, 2012) and other potential selective substrates (Salvi et al., 2012) could allow readouts of PLK2 activity to be examined in PD brain. It would be of interest to determine if the phosphorylation of such substrates correlated to levels of α-synuclein phosphorylation in pathology rich brain regions in PD cases.

### CASEIN KINASES (CKs)

Casein kinases (CKs) comprise a ubiquitously expressed serine/threonine kinase family (Peters et al., 1999) containing two members, CK1 and CK2, which differ substantially in terms of structure, localization and function (Perez et al., 2011). CK1 consists of a small N-terminal lobe, a large C-terminal lobe and a catalytic cleft where ATP and substrates bind (Cheong and Virshup, 2011). To date, at least seven CK1 isoforms (α, β, γ1–3, δ, and ∊) and their various splice variants, ranging from 22 to 55 kDa, have been localized within the membrane, nucleus, and cytoplasm of eukaryote cells, and additionally in the mitotic spindles of mammalian cells (Fish et al., 1995). All CK1 isoforms are highly homologous in their kinase domains (Knippschild et al., 2005), presenting a strong preference for “primed,” pre-phosphorylated substrate. However, they can also phosphorylate related unprimed sites under certain conditions (Cheong and Virshup, 2011).

In contrast to CK1, CK2 is a tetrameric enzyme assembled from two catalytic subunits (CK2α and CK2α′) and a regulatory subunit (CK2β dimer). The two catalytic subunits α and α′ share 90% sequence homology in their N-terminal region, but the regulatory subunit β does not have any similarity to the other two subunits. CK2 is found in many organisms and tissues and nearly every subcellular compartment. It can phosphorylate more than 300 substrate proteins (Meggio and Pinna, 2003) involved in diverse cellular processes including cell division, proliferation, apoptosis, and DNA repair.

Both CK1 and CK2 can constitutively phosphorylate α-synuclein at Ser129 *in vitro* (Okochi et al., 2000; Waxman and Giasson, 2008), and inhibition of CK1 or CK2 reduces α-synuclein Ser129 phosphorylation *in vivo* (Okochi et al., 2000; Ishii et al., 2007; Waxman and Giasson, 2008), with CK2 inhibition seemingly more efficient at reducing phosphorylation (Ishii et al., 2007). However, at least one study failed to find an effect of CK1 inhibition on α-synuclein Ser129 phosphorylation in a cellular model (Waxman and Giasson, 2011). This discrepancy could result from, at least partially, the specificity of CK1 inhibitors and more studies are needed to define the relationship between CKs and α-synuclein.

In pathological human brain CK1δ co-localizes predominantly with tau-containing inclusions such as neurofibrillary tangles, and does not co-localize with α-synuclein in Lewy bodies in PD (Schwab et al., 2000). In contrast, CK2β regulatory subunits are present in the halo region of Lewy bodies in PD substantia nigra (Ryu et al., 2008), suggesting that CK2 may be more pathologically relevant to PD. More work is required to determine any correlations between CK isoforms and the pathological accumulation of phosphorylated α-synuclein in PD.

### G PROTEIN COUPLED RECEPTOR KINASES (GRKs)

G Protein coupled receptor kinases comprise a serine/threonine kinase family that regulate G protein-coupled receptors (GPCRs) by phosphorylating their intracellular domains after their associated G proteins have been released and activated (Gurevich et al., 2012). Structurally, GRKs contain a central catalytic domain flanked by an N-terminus containing a regulator of G protein signaling homology domain and a variable length C-terminal end. Based on sequence homology and tissue expression, GRKs are further classified into three subfamilies: the rhodopsin kinase or visual GRK subfamily (GRK1 and GRK7), the β-adrenergic receptor kinases subfamily (GRK2 and GRK3), and the GRK4 subfamily (GRK4, GRK5, and GRK6; Gurevich et al., 2012; Kamal et al., 2012).

Exactly which GRK isoforms phosphorylate α-synuclein under pathological conditions is unclear. *In vitro* GRK2 preferentially phosphorylates α and β synuclein isoforms while GRK5 prefers α-synuclein as a substrate (Pronin et al., 2000). However, knockdown of either GRK5 or GRK2 failed to diminish the phosphorylation of α-synuclein in cell models (Sakamoto et al., 2009; Liu et al., 2010). In contrast, knockdown of GRK3 or GRK6 significantly decreased α-synuclein Ser129 phosphorylation levels (Sakamoto et al., 2009), suggesting further work is required to verify the role of GRK isoforms in phosphorylating α-synuclein.

G protein coupled receptor kinase isoforms, 2, 3, 5, and 6, are highly expressed in the human brain. In PD brain, however, GRK protein levels tend to be lower than controls (Bychkov et al., 2008) with conflicting reports regarding the co-localization of GRK5 in Lewy bodies (Arawaka et al., 2006; Takahashi et al., 2006).

## OVERVIEW OF KINASES INVOLVED IN α-SYNUCLEIN S129 PHOSPHORYLATION IN PD

Understanding events that promote α-synuclein pathology is increasingly important as evidence suggests a pathogenic prion-like spread of α-synuclein in PD (Olanow and Brundin, 2013; Recasens et al., 2014). There are now multiple human brain tissue studies using the methods developed by Braak and colleagues to observe the progression of pathology in PD brain showing that substantial α-synuclein S129 phosphorylation precedes the aggregation of α-synuclein in Lewy bodies (Zhou et al., 2011; Lue et al., 2012; Walker et al., 2013). Stoichiometrically, PLK2 seemingly contributes most to such α-synuclein S129 phosphorylation; however, studies with PLK2 knockout mice show that other kinases also contribute (Bergeron et al., 2014). Information from other PD models, however, remains controversial on the role α-synuclein S129 phosphorylation plays in disease pathogenesis, with some studies suggesting that S129 phosphorylation promotes α-synuclein oligomerization and/or toxicity (Chen and Feany, 2005; Febbraro et al., 2013) whilst others suggest that phosphorylation reduces toxicity or has no effect (McFarland et al., 2009; Oueslati et al., 2010; Sato et al., 2013; Escobar et al., 2014). This makes determining other relevant kinases difficult and information on any differences between species and models (acute versus chronic) will need further consideration. More data from informative staged human brain studies as well as from primate models with acute and chronic phases is likely to assist with clarifying the role of α-synuclein S129 phosphorylation over the course of PD. It is also important to note that other post-translation modifications of α-synuclein, such as ubiquitylation or nitrosylation, may equally contribute to the pathological process (Oueslati et al., 2010), with similar staged human brain and primate model data on the relative contributions of different protein modifications yet to be published.

## KINASES GENETICALLY IMPLICATED IN PD

Monogenetic causes of PD presently account for less than 10% of all cases (Gasser, 2009). However, the identification of genetic causes has invigorated PD research by providing new avenues of mechanistic investigation and therapeutic treatment. Missense mutations in *LRRK2* and *PINK1* cause PD in an autosomal-dominant or recessive manner, respectively, whilst common variations in the *LRRK2* and loci encoding the *GAK* and *STK39* genes have been implicated as risk factors for PD (Sharma et al., 2012). Understanding how mutations in these kinases alters their function and the biological processes they regulate has great potential for uncovering initiating events leading to the onset of PD.

### LEUCINE-RICH REPEAT KINASE 2 (LRRK2)

Mutations in the *LRRK2* gene were discovered as causal for PD in 2004 (Paisan-Ruiz et al., 2004; Zimprich et al., 2004). Subsequently, some 40 missense mutations have been described across the LRRK2 protein with six of these demonstrated as pathogenic (Paisan-Ruiz, 2009). Collectively these *LRRK2* mutations account for the majority of autosomal-dominantly inherited PD (Kett and Dauer, 2012). *LRRK2*-associated PD is largely clinically and pathologically indistinguishable from sporadic PD (Healy et al., 2008), suggesting that understanding LRRK2 function has implications for all forms of PD. Moreover, large-scale genome-wide association studies show that common variations in non-coding regions of the *LRRK2* gene also confer greater risk for sporadic PD (Satake et al., 2009; Simon-Sanchez et al., 2009; Sharma et al., 2012).

Leucine-rich repeat kinase 2 is a 286 kDa multi-domain containing member of the receptor interacting protein kinase (RIPK) family. LRRK2 has N-terminal ankyrin repeats, leucine-rich repeats, a ras of complex (ROC) GTPase domain with adjoining C-terminal of ROC (COR) domain, a serine/threonine protein kinase domain and C-terminal WD40 repeats. Intriguingly, the majority of the pathogenic mutations lie in the catalytic domains of LRRK2. The most common mutation results in the substitution of glycine to serine (G2019S) in the activation loop of the protein kinase domain resulting in a constitutive threefold increase in LRRK2 kinase activity (West et al., 2005; Jaleel et al., 2007). The next most common mutations, substitution of arginine to either histidine (R1141H), cysteine (R1441C), or glycine (R1441G) lie in the GTPase domain. Some evidence suggests that these mutations also increase kinase activity (Sheng et al., 2012), potentially by trapping LRRK2 in a GTP bound active state (Liao et al., 2014). It has previously been shown that GTP binding is required for LRRK2 kinase activity (Taymans et al., 2011) and a complex relationship exists between the two domains (Taymans, 2012). The increase in catalytic kinase activity with LRRK2 mutations has led to the development of LRRK2 kinase inhibitors as potential PD therapeutics (Deng et al., 2012) and much interest has focused on determining the targets of LRRK2 kinase activity (Dzamko and Halliday, 2013). One such robust effect for LRRK2 kinase activity is to mediate the phosphorylation-dependent interaction of LRRK2 with isoforms of the 14-3-3 adaptor protein (Dzamko et al., 2010); however, consensus regarding the PD-relevant physiological functions of LRRK2 has remained largely elusive.

LRRK2 mRNA expression shows a widespread neuronal localization in human brain; however, intriguingly, only weak levels are detected in the substantia nigra (Higashi et al., 2007; Sharma et al., 2011). Moreover, decreased LRRK2 mRNA was found in certain non-nigral regions of PD brain (cerebellum, amygdala, frontal cortex, and cingulate gyrus; Sharma et al., 2011), suggesting a pathogenic role for LRRK2 outside of nigral neurons. This contrasts with the increased levels of LRRK2 protein reported in PD brain regions with pathological accumulation of α-synuclein (Cho et al., 2013; Guerreiro et al., 2013). The exact nature of the relationship between LRRK2 and α-synuclein is somewhat unclear as postmortem localization studies have produced conflicting results. Some studies have demonstrated localization of LRRK2 to α-synuclein pathology (Miklossy et al., 2006; Alegre-Abarrategui et al., 2008; Qing et al., 2009; Sharma et al., 2011) whilst others have not (Giasson et al., 2006; Higashi et al., 2007; Melrose et al., 2007; Waxman et al., 2009). Studies comparing different LRRK2 antibodies have shown that discrepancies in LRRK2 tissue localization likely occurs through use of antibodies unsuitable for immunohistochemistry (Biskup et al., 2007; Melrose et al., 2007; Davies et al., 2013). Indeed, recent data using more rigorous methods shows LRRK2 and α-synuclein co-localize in a small proportion of PD pathologies (Guerreiro et al., 2013). Despite such data, further work is required to define the relationship between LRRK2 expression and protein levels, between LRRK2 and α-synuclein increases and aggregation, and indeed determine if these proteins interact in the same molecular pathway.

As LRRK2 is also expressed by glial cells in normal human brain (Miklossy et al., 2006) and in tissue culture, and its expression in glia is increased by interferon gamma (Gardet et al., 2010) and bacterial lipopolysaccharide (LPS; Moehle et al., 2012), the neuroinflammation prevalent in PD affected regions may promote the expression changes observed for LRRK2 specifically in microglia rather than neurons. It will also be important to correlate any changes in LRRK2 expression to cell type. The recent demonstration that there is a primate specific LRRK2 promoter that differentiates primate expression of the protein in the brain from that observed in rodents (West et al., 2014) underlies the requirement for further observations in staged human tissue specimens in order to determine the role of LRRK2 kinase function in PD pathogenesis.

### PTEN-INDUCED PUTATIVE KINASE 1 (PINK1)

Homozygous missense mutations in the *PINK1* gene were identified as a cause of familial PD in 2004 (Valente et al., 2004). Around 50 missense mutations have subsequently been identified across the PINK1 protein in a number of populations (Kawajiri et al., 2011). Mutations in *PINK1* are the second most common cause of recessive PD (following mutations in the ubiquitin ligase *Parkin*) and are thought to contribute to 1–8% of familial PD (Kawajiri et al., 2011). Unlike *LRRK2*, *PINK1* mutations reduce kinase activity and cause an atypical form of PD characterized by an early age of onset and slower clinical progression (Abou-Sleiman et al., 2006; Woodroof et al., 2011).

The PINK1 protein comprises a serine/threonine protein kinase domain, a N-terminal mitochondrial targeting motif and a trans-membrane domain located between the two. The mitochondrial targeting motif is required for recruitment of PINK1 to mitochondrial membranes. Following recruitment in healthy mitochondria, PINK1 is enzymatically cleaved to produce a shorter fragment, which is degraded by the proteasome (Narendra et al., 2010). In this way, PINK1 is maintained at very low levels. Pharmacological uncoupling of the mitochondrial membrane leading to a loss of membrane potential; however, results in inhibition of PINK1 cleavage and its accumulation on depolarized mitochondrial membranes (Matsuda et al., 2010; Narendra et al., 2010). The kinase activity of PINK1 is also increased under these conditions with PINK1 undergoing autophosphorylation (Kondapalli et al., 2012). Intriguingly, Parkin is then also recruited to depolarized mitochondria where it is phosphorylated and activated by PINK1 (Matsuda et al., 2010; Kondapalli et al., 2012). Parkin then mediates the degradation of dysfunctional mitochondria by mitophagy (mitochondrial autophagy). Therefore, the two proteins responsible for the majority of familial early onset PD appear to function in the same pathway important for the regulation of mitochondrial quality control. Whether this is the pathway ultimately responsible for loss of neurons in PD is still unclear (Grenier et al., 2013) and it should be noted that PINK1 has also been implicated in other biological processes such as neurite maintenance (Dagda et al., 2014) and inflammation (Lee and Chung, 2012; Kim et al., 2013) among others.

Detailed *in-situ* hybridization studies using rodent brain demonstrate that *PINK1* mRNA is expressed throughout the brain with the strongest signal in neurons of the olfactory bulb, neocortex, prefrontal cortex, piriform cortex, hippocampus, amygdala, brainstem, and cerebellar Purkinje cells (Taymans et al., 2006). Similarly, in human brain, *PINK1* mRNA is widely expressed in neurons with highest signals recorded for the temporal cortex, amygdala, substantia nigra, cerebellar Purkinje cells and the dentate nucleus (Blackinton et al., 2007). *PINK1* mRNA is undetectable in glial cells (Taymans et al., 2006; Blackinton et al., 2007) and is not different in the substantia nigra of sporadic PD patients compared to controls (Blackinton et al., 2007). The mRNA distribution of *PINK1* has been largely confirmed at the protein level with the exception that PINK1 immunoreactivity was also observed in glia, albeit with weak staining compared to neuronal staining (Gandhi et al., 2006). PINK1 is predominantly localized to mitochondria and does not change in amount or localization in the brain of patients with idiopathic PD, although PINK1 immunoreactivity is detected in ~10% of brainstem Lewy bodies (Gandhi et al., 2006). Interestingly, a case report describing the neuropathology of an early onset *PINK1* homozygous mutation patient showed a pattern of Lewy body pathology with atypical Braak Lewy body staging (Samaranch et al., 2010). This was due to the absence of Lewy bodies, and indeed cell loss in the locus coeruleus, potentially helping to explain the longer disease duration of *PINK1*-associated PD (Samaranch et al., 2010). Collectively these studies suggest that alterations in PINK1 function, rather than protein levels, likely contribute to PD. This is consistent with observations that the majority of described *PINK1* mutations result in a loss of kinase activity (Woodroof et al., 2011). Whether PINK1 autophosphorylation or PINK1-induced Parkin phosphorylation are altered in sporadic PD, and how this correlates to mitochondrial health and/or neuronal loss, would be interesting to explore.

### CYCLIN G-ASSOCIATED KINASE (GAK)

The Ser/Thr protein kinase GAK was originally identified via its interaction with cyclin G and cyclin-dependent kinase 5 (CDK5; Kanaoka et al., 1997). The kinase domain is located at the N-terminus and a leucine-zipper region is located at the C-terminus. The majority of the protein comprises a TAG domain that has 80% identity to the auxilin protein (Kanaoka et al., 1997). Both auxilin and GAK play key roles in the uncoating of clatherin-coated vesicles and the regulation of clatherin-mediated endocytosis (Eisenberg and Greene, 2007). The latter is completely blocked in GAK deficient mouse embryonic fibroblasts (Lee et al., 2008). GAK also plays a key role in brain development. Conditional deletion of GAK in mouse brain resulted in marked cell loss and morphological changes in new-born pups, potentially due to a lack of proliferation of neural progenitor cells in the subventricular zone of the hippocampus. Conditional GAK knockout mice die soon after birth whilst conventional GAK knockouts are embryonic lethal (Lee et al., 2008). Moreover transgenic mice expressing kinase inactive GAK die within 30 min of birth due to respiratory dysfunction (Tabara et al., 2011). Respiratory problems are also associated with use of gefitinib (Tabara et al., 2011), an anti-cancer epidermal growth factor receptor (EGFR) inhibitor that also inhibits GAK.

Single nucleotide polymorphisms in the *GAK* locus were first associated with PD susceptibility following genome-wide association analysis of a large number of familial PD patients (Pankratz et al., 2009). The association has since been robustly replicated in different populations (Rhodes et al., 2011; Sharma et al., 2012). One *GAK* SNP, rs1564282 is associated with higher expression of α-synuclein in PD brain, and when *GAK* mRNA was knocked down with siRNA, there was accumulation of α-synuclein in cell culture models (Dumitriu et al., 2011). This provides some biochemical evidence for a role for GAK in PD, although the toxic effects of GAK knockdown/inhibition suggest that GAK is unlikely to be dramatically decreased in PD brain. Moreover, an alternative microarray based study has shown that *GAK* mRNA expression is increased in the substantia nigra of PD patients (Grunblatt et al., 2004). Intriguingly, GAK has also been proposed to interact with LRRK2 and potentially help co-ordinate the clearance of trans-Golgi derived vesicles (Beilina et al., 2014); however, GAK protein expression in PD brain and any association with PD pathology has been poorly explored.

### SERINE/THREONINE KINASE 39 (STK39)

Serine/threonine kinase is more commonly referred to in the literature as SPAK. The majority of work on SPAK has focused on the enzymes role as a regulator of the Na^+^/Cl^-^ and Na^+^/K^+^/2Cl^-^ ion co-transporters, NCC and NKCC, respectively. In response to osmotic stress SPAK is activated by phosphorylation at T233 in its activation loop by isoforms of WNK (with-no lysine) kinases and in turn phosphorylates NCC/NKCC to promote transporter activity (Richardson and Alessi, 2008). These ion co-transporters are major drug targets of current anti-hypertensive medications and evidence suggests that inhibition of SPAK may also lower blood pressure (Richardson and Alessi, 2008; Glover and O’shaughnessy, 2011). Indeed, variations in the *STK39* gene have been implicated in hypertension in the Amish population through genome-wide association, with the resulting non-coding mutations increasing the allelic expression of SPAK (Wang et al., 2009a). This association, however, has failed to reach significance in other populations (Cunnington et al., 2009; Persu and Vikkula, 2011).

Genome-wide association studies have also implicated variations in the *STK39* locus with PD. First identified through large-scale meta-analysis (Liu et al., 2011; Nalls et al., 2011), the association of *STK39* SNPs with PD has been subsequently replicated in Asian and Caucasian populations (Lill et al., 2012; Sharma et al., 2012). The three reported *STK39* SNPs associating with PD differ from the reported SNP for hypertension. Whether these polymorphisms affect SPAK expression is unknown.

Studies using rats show that SPAK is highly expressed in the nervous system, especially brain where it is detected in neurons, Purkinje cells and choroid epithelial cells (Ushiro et al., 1998). Glial cells do not show immunoreactivity for SPAK (Ushiro et al., 1998). In developing brain, SPAK plays a role in the regulation of Cl^-^ concentration and in-turn release of the neurotransmitter GABA (Delpire and Austin, 2010). SPAK has also been suggested to act as a stress-response kinase with its overexpression or activation leading to increased phosphorylation of p38 MAPK (Yan et al., 2007). Whether levels of SPAK protein, phosphorylation of SPAK T233 or phosphorylation of the SPAK ion transporter substrates are altered in PD brain has not been investigated.

## OVERVIEW OF KINASES GENETICALLY IMPLICATED IN PD

There is much interest in LRRK2 as both a key to understanding PD pathogenesis and a potential therapeutic target, as *PINK1* mutations cause an atypical form of PD and the mechanism/s of increased PD risk due to *SPAK* and *GAK* polymorphisms is presently unclear. The mRNA expression of *LRRK2* is decreased in PD brain; however, LRRK2 protein is increased, at least in Lewy body-rich regions at end-stage disease. Further work is required to determine if LRRK2 protein is altered earlier in PD pathogenesis and in particular, as LRRK2 expression can be induced with inflammatory agonists in microglia (Moehle et al., 2012), the cell types expressing LRRK2 may be important. Localization studies of LRRK2, and indeed PINK1 have proven difficult, as a number of available antibodies are not optimal for this procedure. Moreover, the kinase activity of LRRK2 has not been explored in PD brain. This is potentially important as kinase inhibiting therapeutics are being targeted toward LRRK2, even though it is unclear if the toxic effects of LRRK2 mutations are kinase-dependent. At least one risk variant reportedly decreases LRRK2 kinase activity (Rudenko et al., 2012b) leading to suggestions that other functions of LRRK2 such as GTPase activity may be important (Rudenko et al., 2012a). The identification of *bona fide* substrates for PINK1 and LRRK2 will be important for inferring any changes in enzymatic activity in the PD brain.

## KINASES EXPERIMENTALLY IMPLICATED IN PD

### KINASES OF THE MITOGEN ACTIVATED PROTEIN KINASE (MAPK) PATHWAY

**FIGURE 2:**
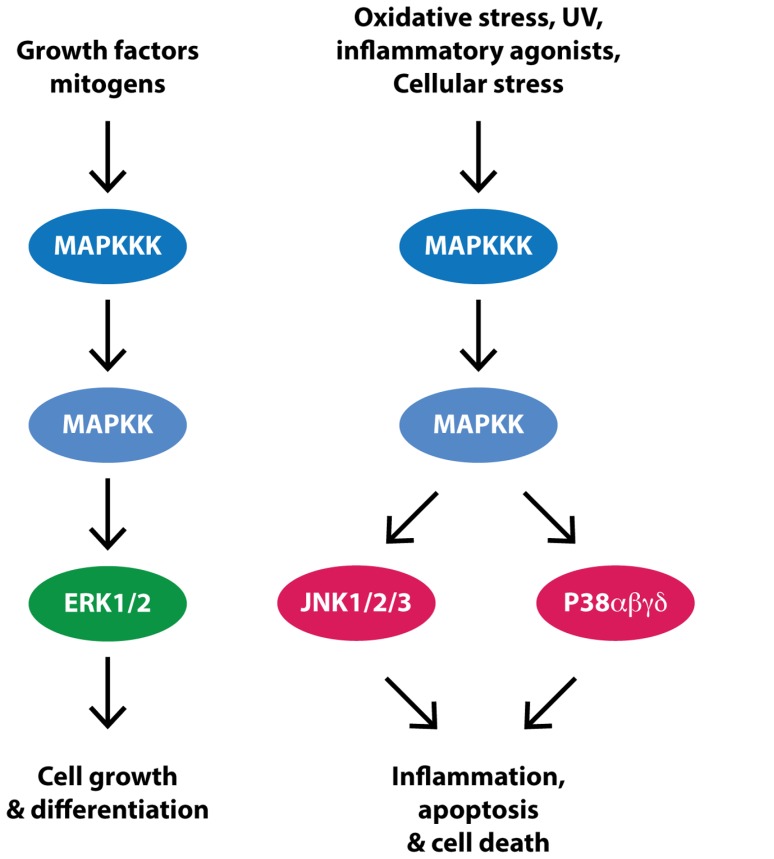
**Simplifed MAPK signaling**. Receptor activation by growth factors or mitogens triggers a signal cascade in which mitogen activated protein kinase kinase kinases (MAPKKK) are activated and in turn activate mitogen activated kinase kinases (MAPKK) and then the mitogen activated protein kinases (MAPK), ERK, JNK, and P38. Evidence suggests that this pathway is upregulated in PD substantia nigra dopaminergic neurons, potentially contributing to cell death.

The MAPK superfamily of serine/threonine protein kinases consists of three major branches, the JNKs, the p38 kinases and the ERKs (**Figure 2**; for review see Kyriakis and Avruch, 2012). The three JNK isoforms (JNK1, JNK2, and JNK3) and four p38 isoforms (p38α, p38β, p38γ, and p38δ) are referred to as stress activated protein kinases (SAPKs). In particular, JNK is activated by a number of environmental stresses implicated in PD including, toxins, inflammatory agonists and misfolded protein-induced ER stress. The activation of p38 is more restricted to inflammatory agonists whilst the two ERK isoforms (ERK1 and ERK2) are activated principally in response to mitogens, although a high level of cross-talk exists between the different MAPK branches. Upon activation, JNK, ERK, and p38 phosphorylate a large number of substrates in a proline-directed manner. In some instances substrates can be specific, such as JNK to phosphorylate the AP-1 transcription factor component c-Jun, or ERK to phosphorylate the p90 ribosomal S6 kinase (RSK), or shared, such as ERK and p38 to phosphorylate the mitogen and stress activated kinase (MSK). Biologically, the MAPKs modulate a number of important functions including development, immunity, apoptosis, cell growth and division, autophagy and cell survival.

#### JNK and p38

c-Jun N-terminal kinase is robustly activated in common toxin models of PD such as LPS, 1-methyl-4-phenyl-1,2,3,6-tetrahydropyridine (MPTP) and 6-hydroxydopamine (6-OHDA; Choi et al., 1999; Saporito et al., 2000; Xing et al., 2007). Genetic deletion of JNK2 and JNK3 protect against MPTP-induced neurodegeneration in mice (Hunot et al., 2004) and kinase inhibitors of JNK have neuroprotective effects in the MPTP (Saporito et al., 1999; Wang et al., 2004, 2009b; Chambers et al., 2011) and 6-OHDA (Chambers et al., 2013) models of PD. Moreover a host of anti-oxidant and anti-inflammatory compounds offering varying degrees of neuroprotection in these models are thought to have a mechanism of action, at least in part, involving inhibition of JNK activation (Xing et al., 2007; Castro-Caldas et al., 2012; Lee et al., 2013; Zhai et al., 2013). Genetic deletion of the p38 substrate MK2, also protects against MPTP-induced neurodegeneration in rodents, by reducing the neuroinflammation associated with MPTP lesions (Thomas et al., 2008). Both JNK and p38 are also implicated in the death of neuronal cells following treatment with another environmental toxin used to model PD, rotenone (Newhouse et al., 2004; Gao et al., 2013), with inhibition of p38 potentially protective (Choi et al., 2014). Despite the evidence from cellular and animal models, however, a clinical trial of the JNK inhibitor CEP-1347 failed to show benefit in human PD patients (Investigators, 2007), possibly because of an absence of substantial changes in these kinases in patients with chronic PD (Ferrer et al., 2001).

Increased nuclear staining of the JNK substrate, c-Jun, has been observed in the substantia nigra of PD patients (Hunot et al., 2004). Translocation of c-Jun to the nucleus requires JNK phosphorylation and is a surrogate marker of JNK activity. The association between JNK and p38 and α-synuclein pathology has also been explored in the substantia nigra and brainstem regions of control and PD brain. In this study, granular phosphorylated p38 immunoreactivity was observed in association with diffuse α-synuclein pathology, more consistent with Lewy neurites than Lewy bodies in the substantia nigra (Ferrer et al., 2001). In contrast, phosphorylated JNK rarely stained Lewy body containing neurons (Ferrer et al., 2001). There was also no association between phosphorylated JNK immunostaining and apoptosis in PD substantia nigra neurons (Ferrer et al., 2001). These studies suggest a potential early role for p38 in the formation of Lewy bodies whereas JNK appears not to be involved. The protective effects of JNK inhibitors may instead be mediated through glial cells. Further studies could explore how JNK activity in glia relates to PD pathogenesis.

#### ERK

Extracellular signal related protein kinase is activated following treatment of cells with 6-OHDA and MPTP and inhibitors of ERK provide protection in these PD cell models (Kulich and Chu, 2001; Gomez-Santos et al., 2002). ERK crosstalk also modulates protective effects of neurotrophins and anti-oxidant treatments (Chu et al., 2004; Hetman and Gozdz, 2004).

In neurons in the substantia nigra of PD patients, phosphorylated ERK immunoreactivity shows granular aggregations, distinct from the diffuse cytoplasmic localization of phosphorylated ERK in cortical neurons from control and PD patients (Zhu et al., 2002). The aggregated pattern of phosphorylated ERK staining is also observed in pigmented neurons of the locus coeruleus in PD patients, but is absent in glial cells. The levels of phosphorylated ERK increase in substantia nigra neurons in PD patients, where the granular inclusions partly associate with mitochondria and weakly with endosomes (Zhu et al., 2003). Increased phosphorylation of ERK correlates with increased staining for the ERK substrate RSK1 (Zhu et al., 2002) and total levels of ERK do not differ between control and PD samples (Zhu et al., 2002), collectively demonstrating an increase in ERK activity in PD brain. ERK also associates with Lewy bodies, particularly the halo region (Ferrer et al., 2001; Zhu et al., 2002, 2003). Moreover, granular ERK inclusions are often seen in PD neurons devoid of α-synuclein pathology and sometimes not seen at all in neurons with severe α-synuclein pathology suggesting a potential early role for ERK in PD pathogenesis (Zhu et al., 2002).

### KINASES OF THE PHOSPHOINOSITIDE 3-KINASE (PI3K) PATHWAY

**FIGURE 3:**
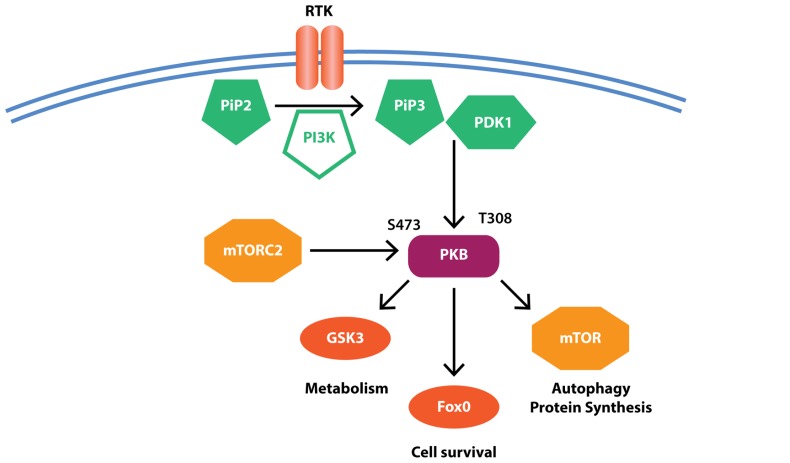
**Simplified PI3K signaling**. Receptor-ligand binding results in the activation of phosphoinositide 3-kinase (PI3K) that in turn mediates the conversion of phosphatidylinositol 4,5-bisphosphate (PIP2) to phosphatidylinositol 2,4,5-triphosphate (PIP3). PIP3 recruits protein kinase B (PKB) where it is activated by phosphorylation at Thr308 by phosphoinositide-dependent kinase 1 (PDK1) and Ser473 by mammalian target of rapamycin (mTOR) complex 2 (mTORC2). PKB then further phosphorylates downstream substrates to regulate cell survival and metabolic pathways. Evidence suggests reduced PKB but increased mTOR and GSK3β activity in PD, potentially contributing to protein accumulation and reduced cell survival.

The PI3K pathway controls cell survival and proliferation and thus has been studied extensively in the context of cancer. PI3K is classically activated by tyrosine kinase receptors following their binding of insulin or insulin like growth factors (e.g., IGF1; **Figure 3**). Activated PI3K phosphorylates membrane-associated phosphatidylinositol 4,5-bisphosphate (PIP2) to produce phosphatidylinositol 2,4,5-triphosphate (PIP3), which results in recruitment of PKB (also known as AKT; **Figure 3**). PKB is in turn phosphorylated in its activation loop (at Thr308) by phosphoinositide-dependent kinase 1 (PDK1) and at its C-terminal hydrophobic motif (Ser473) by mTOR complex 2 (mTORC2). Activated PKB phosphorylates a number of substrates including mTOR, to promote protein synthesis and inhibit autophagy, glycogen synthase kinase 3β (GSK3β), to induce glycogen and regulate glucose metabolism and fork head box-O class (FOXO), a transcription factor regulating genes essential for cell growth, proliferation, and survival.

#### PKB

Administration of 6-OHDA results in reduced PKB Thr308 and Ser473 phosphorylation and marked loss of PKB activity in cell culture and rodent PD models and a number of compounds that stimulate PKB activity have demonstrated neuroprotection in this model, as well as other PD toxin models, including MPTP and rotenone (for review see Greene et al., 2011). Overexpression of PKB in rodent brain also protects dopaminergic neurons from 6-OHDA-induced cell death (Ries et al., 2006) and activation of PKB likely contributes to the neuroprotective effects of trophic factors such as glial cell line-derived neurotrophic factor (GDNF; Ugarte et al., 2003) and potentially to the effects of the monoamine oxidase B inhibitor and PD drug rasagiline (Mandel et al., 2007; Sagi et al., 2007).

In PD midbrain the phosphorylation of PKB Ser473 is reduced in both cytosolic and membrane fractions (Timmons et al., 2009). PKB and its phosphorylation at Ser473 are also robustly detected in dopaminergic neurons of the substantia nigra and are consequently reduced in PD with loss of these neurons. However, PKB immunoreactivity is still detected in surviving PD dopaminergic neurons (Timmons et al., 2009). A second study has confirmed reduced phosphorylation of PKB at both Thr308 and Ser473 in PD substantia nigra dopaminergic neurons (Malagelada et al., 2008). Results of this study suggest that reduced phosphorylation of PKB is restricted to dopaminergic neurons as non-neuromelanin containing neurons of the midbrain expressed similar levels of PKB and phosphorylated PKB in both control and PD states. Interestingly, a robust increase in PKB and phosphorylated Ser473 PKB was detected in cells with glial morphology in the substantia nigra region in PD (Timmons et al., 2009). Whilst reduced PKB pathway activity in neurons may contribute to their loss in PD, the contribution of increased PKB activity in glia to the progression of PD has not been explored.

#### GSK3β

The two isoforms of GSK3, GSK3α, and GSK3β, are ubiquitously expressed in the brain where they predominantly act to regulate glucose metabolism. Inhibition of the GSK3β isoform can protect against MPTP, 6-OHDA, and LPS-induced neurotoxicity (Kozikowski et al., 2006; Wang et al., 2007; Morales-Garcia et al., 2013) whilst its activation has been implicated in rotenone toxicity (Hongo et al., 2012). GSK3β has also been implicated in microglial-mediated inflammation (Yuskaitis and Jope, 2009) and the neuroprotective effects of GSK3β inhibitors may be mediated, at least in part, through anti-inflammatory actions (Yuskaitis and Jope, 2009; Morales-Garcia et al., 2013).

In brain, increased total and phosphorylated GSK3β is detected as punctate structures in the cytosol of pigmented neurons in PD substantia nigra (Nagao and Hayashi, 2009). GSK3β and phosphorylated GSK3β partly co-localize to the halo region of Lewy bodies and also to Lewy neurites (Nagao and Hayashi, 2009). GSK3β protein is also significantly increased in the striatum of PD brains where its phosphorylation correlates with both tau and α-synuclein pathology (Wills et al., 2010). This suggests a potential role for GSK3β in promoting the early stages of tau interaction with α-synuclein, leading to α-synuclein pathology in PD. This could be important as genome-wide association studies implicate polymorphisms in the *MAPT* and *SNCA* genes as the most robustly reproducible risk factors for sporadic PD (Satake et al., 2009; Simon-Sanchez et al., 2009).

#### mTOR

The mTOR kinase exists in two complexes termed mTORC1 and mTORC2, with mTORC2 regulating PKB activity and mTORC1 regulating protein synthesis and autophagy (Laplante and Sabatini, 2012). The phosphorylation of mTORC1 by PKB promotes protein synthesis and inhibits autophagy. Reduced phosphorylation of PKB in PD brain may therefore be expected to promote autophagy; however, this process is clearly dysregulated in PD as autophagy markers are also significantly decreased in PD substantia nigra (Chu et al., 2009; Alvarez-Erviti et al., 2010). Indeed, evidence suggests that dysfunctional autophagy pathways play a key role in the pathogenesis of PD (Lynch-Day et al., 2012). Moreover, the mTOR inhibitor (and therefore autophagy inducing) rapamycin prevents MPTP-induced neurodegeneration (Dehay et al., 2010; Liu et al., 2013). Rapamycin also has protective properties in rotenone and α-synuclein PD models (Pan et al., 2009; Spencer et al., 2009; Crews et al., 2010; Xiong et al., 2011) suggesting inhibition of mTORC1 has potential as a treatment for PD.

Protein levels of neuronal mTOR were significantly increased in the temporal cortex of cases with dementia with Lewy bodies, particularly in neurons displaying accumulation of α-synuclein (Crews et al., 2010). In comparison, brain tissue from cases with Alzheimer’s disease had normal levels of mTOR in the temporal cortex (Crews et al., 2010). Up-regulation of mTOR is consistent with a phenotype of increased protein synthesis and reduced autophagy, promoting the accumulation of potentially toxic proteins. In the context of PD, increased mTOR would likely aid the propagation of α-synuclein, however, whether changes in mTOR are associated with the spread of α-synuclein pathology in PD brain is unknown.

## OVERVIEW OF KINASES EXPERIMENTALLY IMPLICATED IN PD

It is evident from human tissue studies, particularly those focused on the nigral dopaminergic system, that inflammatory pathways are activated in PD and autophagy pathways are impaired. PD brain tissue samples from different brain regions at different stages of pathology could inform on the order of these events and provide more insight into whether certain kinases are causal for PD pathologies. The discovery that certain toxins (MPTP, rotenone, LPS, 6-OHDA) induce a selective loss of dopaminergic neurons in rodent models has facilitated a wealth of information regarding the order of the biological processes leading to such neuronal death as well as signaling proteins mediating these events. While these toxin-based models do not replicate all the features of sporadic PD, such as an age-dependent phenotype and the presence of α-synuclein pathology, they have implicated a range of kinases as important in the process. It will be important in many instances to use pathologically staged human brain tissue to validate the expression of kinases and their isoforms and any disease-associated changes identified experimentally in mice.

## ADDITIONAL KINASES AND OVERALL CONCLUSIONS

In addition to the kinases discussed above, a number of other kinases are emerging as having potential roles in PD pathogenesis and/or potential therapeutic targets. These include CDK5, a kinase whose activity is increased by MPTP treatment and inhibition attenuates MPTP-induced neuronal loss (Smith et al., 2003; Qu et al., 2007). The eIF2alpha kinase (also known as PERK), whose inhibition was recently shown to attenuate neurodegeneration in prion-infected mice (Moreno et al., 2013), and AMP-activated protein kinase (AMPK), a major metabolic regulatory enzyme whose activation has been associated with neuroprotection in a number of PD models (Wu et al., 2011; Bayliss and Andrews, 2013; Dulovic et al., 2014; Li et al., 2014). AMPK is also activated by thiazolidinones (Fryer et al., 2002), compounds that have neuroprotective properties in a number of settings (Carta, 2013). Of these kinases, CDK5 and PERK have been studied in PD brain, with CDK5 localizing to Lewy bodies (Brion and Couck, 1995; Nakamura et al., 1997) and PERK increased in PD substantia nigra neurons (Hoozemans et al., 2007). PERK levels also correlated with α-synuclein deposition (Hoozemans et al., 2007), making PERK in particular a very interesting candidate for further study.

**FIGURE 4:**
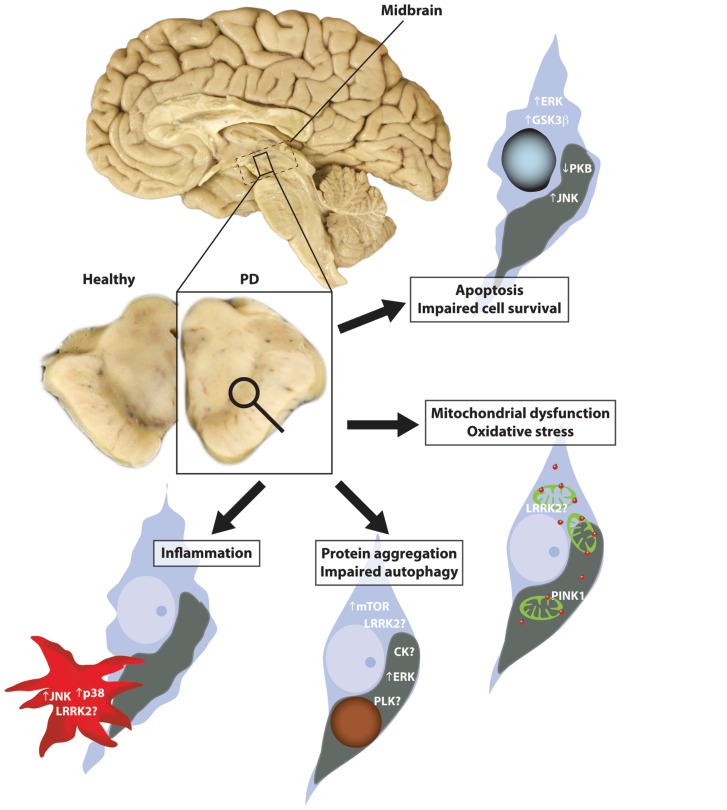
**Kinases implicated in Parkinson’s disease (PD)**. PD is characterized by the loss of pigmented dopaminergic neurons in the substantia nigra region of the midbrain. A number of biological processes have been implicated in this neuronal loss including mitochondrial dysfunction, oxidative stress, autophagy, and inflammation. In the substantia nigra, a number of kinases impacted by these processes combine to promote the accumulation of phosphorylated α -synuclein and induce conditions that reduce cell viability.

Thus, a number of protein kinases have been implicated in the pathogenesis of PD covering a diverse array of biological functions including oxidative stress, inflammation, and autophagy (**Figure 4**). However, delineating the exact order by which these biological functions go wrong in PD brain is still a major challenge, despite the staging methods now in more common use. It is likely that some kinases are more important for initiating the disease whilst others are more important for disease propagation. In this regard, the majority of brain tissue work to date has focused on the substantia nigra region in PD, a region mostly at end-stage pathology in patients dying with PD. Thus brain tissue studies using this region are not informative on early pathogenic events, and assessment of this region provides limited information on the cause or consequence of many findings. With a greater understanding of how PD spreads throughout the brain in a staged fashion, brain regions can be selected to determine biochemical responses across the disease spectrum, particularly assessing regions with evidence of only early perturbations indicative of PD. Such an approach should provide some insight into which processes may precede PD pathology and which processes may propagate PD pathology. This is important for determining when potential therapies, such as kinase inhibitors, are likely to exert maximum efficacy. It is also important to recognize potential caveats of postmortem studies such as postmortem delay, comorbidities, and drug regimes, even though many caveats can be controlled with appropriate sample selection. In the absence of animal models that replicate all the cardinal features of PD, human pathological postmortem brain tissue remains an important resource to understand the biochemical details of PD and to verify cell and animal model hypothesis testing.

## Conflict of Interest Statement

The authors declare that the research was conducted in the absence of any commercial or financial relationships that could be construed as a potential conflict of interest.

## References

[B1] Abou-Sleiman P. M. Muqit M. M. Wood N. W. (2006). Expanding insights of mitochondrial dysfunction in Parkinson’s disease. *Nat. Rev. Neurosci.* 7 207–219 10.1038/nrn1868 16495942

[B2] Alegre-Abarrategui J. Ansorge O. Esiri M. Wade-Martins R. (2008). LRRK2 is a component of granular alpha-synuclein pathology in the brainstem of Parkinson’s disease. *Neuropathol. Appl. Neurobiol.* 34 272–283 10.1111/j.1365-2990.2007.00888.x 17971075 PMC2833010

[B3] Alvarez-Erviti L. Rodriguez-Oroz M. C. Cooper J. M. Caballero C. Ferrer I. Obeso J. A. (2010). Chaperone-mediated autophagy markers in Parkinson disease brains. *Arch. Neurol.* 67 1464–1472 10.1001/archneurol.2010.198 20697033

[B4] Anderson J. P. Walker D. E. Goldstein J. M. De Laat R. Banducci K. Caccavello R. J. (2006). Phosphorylation of Ser-129 is the dominant pathological modification of alpha-synuclein in familial and sporadic Lewy body disease. *J. Biol. Chem.* 281 29739–29752 10.1074/jbc.M600933200 16847063

[B5] Arawaka S. Wada M. Goto S. Karube H. Sakamoto M. Ren C. H. (2006). The role of G-protein-coupled receptor kinase 5 in pathogenesis of sporadic Parkinson’s disease. *J. Neurosci.* 26 9227–9238 10.1523/JNEUROSCI.0341-06.2006 16957079 PMC6674490

[B6] Bayliss J. A. Andrews Z. B. (2013). Ghrelin is neuroprotective in Parkinson’s disease: molecular mechanisms of metabolic neuroprotection. *Ther. Adv. Endocrinol. Metab.* 4 25–36 10.1177/2042018813479645 23515333 PMC3593299

[B7] Beilina A. Rudenko I. N. Kaganovich A. Civiero L. Chau H. Kalia S. K. (2014). Unbiased screen for interactors of leucine-rich repeat kinase 2 supports a common pathway for sporadic and familial Parkinson disease. *Proc. Natl. Acad. Sci. U.S.A.* 111 2626–2631 10.1073/pnas.1318306111 24510904 PMC3932908

[B8] Bergeron M. Motter R. Tanaka P. Fauss D. Babcock M. Chiou S.S. (2014). In vivo modulation of polo-like kinases supports a key role for PLK2 in Ser129 alpha-synuclein phosphorylation in mouse brain. *Neuroscience* 256 72–82 10.1016/j.neuroscience.2013.09.061 24128992

[B9] Biskup S. Moore D. J. Rea A. Lorenz-Deperieux B. Coombes C. E. Dawson V. L. (2007). Dynamic and redundant regulation of LRRK2 and LRRK1 expression. *BMC Neurosci.* 8:102 10.1186/1471-2202-8-102 PMC2233633 18045479

[B10] Blackinton J. G. Anvret A. Beilina A. Olson L. Cookson M. R. Galter D. (2007). Expression of PINK1 mRNA in human and rodent brain and in Parkinson’s disease. *Brain Res.* 1184 10–16 10.1016/j.brainres.2007.09.056 17950257

[B11] Bowers S. Truong A. P. Ye M. Aubele D. L. Sealy J. M. Neitz R. J. (2013). Design and synthesis of highly selective, orally active Polo-like kinase-2 (Plk-2) inhibitors. *Bioorg. Med. Chem. Lett.* 23 2743–2749 10.1016/j.bmcl.2013.02.065 23522834

[B12] Braak H. Del Tredici K. Rub U. De Vos R. A. Jansen Steur E. N. Braak E. (2003). Staging of brain pathology related to sporadic Parkinson’s disease. *Neurobiol. Aging* 24 197–211 10.1016/S0197-4580(02)00065-9 12498954

[B13] Brion J. P. Couck A. M. (1995). Cortical and brainstem-type Lewy bodies are immunoreactive for the cyclin-dependent kinase 5. *Am. J. Pathol.* 147 1465–1476 7485409 PMC1869502

[B14] Bychkov E. R. Gurevich V. V. Joyce J. N. Benovic J. L. Gurevich E. V. (2008). Arrestins and two receptor kinases are upregulated in Parkinson’s disease with dementia. *Neurobiol. Aging* 29 379–396 10.1016/j.neurobiolaging.2006.10.012 17125886 PMC2275668

[B15] Carlsson A. (1959). The occurrence, distribution and physiological role of catecholamines in the nervous system. *Pharmacol. Rev.* 11 490–493 13667431

[B16] Carta A. R. (2013). PPAR-gamma: therapeutic prospects in Parkinson’s disease. *Curr. Drug Targets* 14 743–751 10.2174/1389450111314070004 23469878

[B17] Castro-Caldas M. Carvalho A. N. Rodrigues E. Henderson C. J. Wolf C. R. Rodrigues C. M. (2012). Tauroursodeoxycholic acid prevents MPTP-induced dopaminergic cell death in a mouse model of Parkinson’s disease. *Mol. Neurobiol.* 46 475–486 10.1007/s12035-012-8295-4 22773138

[B18] Chambers J. W. Howard S. Lograsso P. V. (2013). Blocking c-Jun N-terminal kinase (JNK) translocation to the mitochondria prevents 6-hydroxydopamine-induced toxicity in vitro and in vivo. *J. Biol. Chem.* 288 1079–1087 10.1074/jbc.M112.421354 23184940 PMC3542993

[B19] Chambers J. W. Pachori A. Howard S. Ganno M. Hansen D. Jr. Kamenecka T. (2011). Small molecule c-jun-N-terminal kinase (JNK) inhibitors protect dopaminergic neurons in a model of Parkinson’s disease. *ACS Chem. Neurosci.* 2 198–206 10.1021/cn100109k 21666839 PMC3110074

[B20] Chen L. Feany M. B. (2005). Alpha-synuclein phosphorylation controls neurotoxicity and inclusion formation in a *Drosophila* model of Parkinson disease. *Nat. Neurosci.* 8 657–663 10.1038/nn1443 15834418

[B21] Cheong J. K. Virshup D. M. (2011). Casein kinase 1: complexity in the family. *Int. J. Biochem. Cell Biol.* 43 465–469 10.1016/j.biocel.2010.12.004 21145983

[B22] Cho H. J. Liu G. Jin S. M. Parisiadou L. Xie C. Yu J. (2013). MicroRNA-205 regulates the expression of Parkinson’s disease-related leucine-rich repeat kinase 2 protein. *Hum. Mol. Genet.* 22 608–620 10.1093/hmg/dds470 23125283 PMC3542867

[B23] Choi B. S. Kim H. Lee H. J. Sapkota K. Park S. E. Kim S. (2014). Celastrol from ‘Thunder God Vine’ protects SH-SY5Y cells through the preservation of mitochondrial function and inhibition of p38 MAPK in a rotenone model of Parkinson’s disease. *Neurochem. Res.* 39 84–96 10.1007/s11064-013-1193-y 24214023

[B24] Choi W. S. Yoon S. Y. Oh T. H. Choi E. J. O’malley K. L. Oh Y. J. (1999). Two distinct mechanisms are involved in 6-hydroxydopamine- and MPP^+^-induced dopaminergic neuronal cell death: role of caspases, ROS, and JNK. *J. Neurosci. Res.* 57 86–94 10.1002/(SICI)1097-4547(19990701)57:1<86::AID-JNR9>3.0.CO;2-E 10397638

[B25] Chu C. T. Levinthal D. J. Kulich S. M. Chalovich E. M. Defranco D. B. (2004). Oxidative neuronal injury. The dark side of ERK1/2. *Eur. J. Biochem*. 271 2060–2066 10.1111/j.1432-1033.2004.04132.x 15153095 PMC1899467

[B26] Chu Y. Dodiya H. Aebischer P. Olanow C. W. Kordower J. H. (2009). Alterations in lysosomal and proteasomal markers in Parkinson’s disease: relationship to alpha-synuclein inclusions. *Neurobiol. Dis.* 35 385–398 10.1016/j.nbd.2009.05.023 19505575

[B27] Crews L. Spencer B. Desplats P. Patrick C. Paulino A. Rockenstein E. (2010). Selective molecular alterations in the autophagy pathway in patients with Lewy body disease and in models of alpha-synucleinopathy. *PLoS ONE* 5:e9313 10.1371/journal.pone.0009313 PMC2824828 20174468

[B28] Cunnington M. S. Kay C. Avery P. J. Mayosi B. M. Koref M. S. Keavney B. (2009). STK39 polymorphisms and blood pressure: an association study in British Caucasians and assessment of cis-acting influences on gene expression. *BMC Med. Genet.* 10:135 10.1186/1471-2350-10-135 PMC2803166 20003416

[B29] Dagda R. K. Pien I. Wang R. Zhu J. Wang K. Z. Callio J. (2014). Beyond the mitochondrion: cytosolic PINK1 remodels dendrites through protein kinase A. *J. Neurochem.* 128 864–877 10.1111/jnc.12494 24151868 PMC3951661

[B30] Davies P. Hinkle K. M. Sukar N. N. Sepulveda B. Mesias R. Serrano G. (2013). Comprehensive characterization and optimization of anti-LRRK2 (leucine-rich repeat kinase 2) monoclonal antibodies. *Biochem. J.* 453 101–113 10.1042/BJ20121742 23560750 PMC3682752

[B31] de Carcer G. Escobar B. Higuero A. M. Garcia L. Anson A. Perez G. (2011a). Plk5, a polo box domain-only protein with specific roles in neuron differentiation and glioblastoma suppression. *Mol. Cell. Biol.* 31 1225–1239 10.1128/MCB.00607-10 21245385 PMC3067912

[B32] de Carcer G. Manning G. Malumbres M. (2011b). From Plk1 to Plk5: functional evolution of polo-like kinases. *Cell Cycle* 10 2255–2262 10.4161/cc.10.14.16494 21654194 PMC3230524

[B33] Dehay B. Bove J. Rodriguez-Muela N. Perier C. Recasens A. Boya P. (2010). Pathogenic lysosomal depletion in Parkinson’s disease. *J. Neurosci.* 30 12535–12544 10.1523/JNEUROSCI.1920-10.2010 20844148 PMC6633458

[B34] Delpire E. Austin T. M. (2010). Kinase regulation of Na^+^–K^+^–2Cl^-^ cotransport in primary afferent neurons. *J. Physiol.* 588 3365–3373 10.1113/jphysiol.2010.190769 20498230 PMC2988503

[B35] Deng X. Choi H. G. Buhrlage S. J. Gray N. S. (2012). Leucine-rich repeat kinase 2 inhibitors: a patent review (2006–2011). *Expert Opin. Ther. Pat.* 22 1415–1426 10.1517/13543776.2012.729041 23126385

[B36] Dulovic M. Jovanovic M. Xilouri M. Stefanis L. Harhaji-Trajkovic L. Kravic-Stevovic T. (2014). The protective role of AMP-activated protein kinase in alpha-synuclein neurotoxicity in vitro. *Neurobiol. Dis.* 63 1–11 10.1016/j.nbd.2013.11.002 24269733

[B37] Dumitriu A. Pacheco C. D. Wilk J. B. Strathearn K. E. Latourelle J. C. Goldwurm S. (2011). Cyclin-G-associated kinase modifies alpha-synuclein expression levels and toxicity in Parkinson’s disease: results from the GenePD Study. *Hum. Mol. Genet.* 20 1478–1487 10.1093/hmg/ddr026 21258085 PMC3063983

[B38] Dzamko N. Deak M. Hentati F. Reith A. D. Prescott A. R. Alessi D. R. (2010). Inhibition of LRRK2 kinase activity leads to dephosphorylation of Ser(910)/Ser(935), disruption of 14-3-3 binding and altered cytoplasmic localization. *Biochem. J.* 430 405–413 10.1042/BJ20100784 20659021 PMC3631100

[B39] Dzamko N. Halliday G. M. (2013). Unlocking the secrets of LRRK2 function with selective kinase inhibitors. *Future Neurol.* 8 347–357 10.2217/fnl.13.9

[B40] Eisenberg E. Greene L. E. (2007). Multiple roles of auxilin and hsc70 in clathrin-mediated endocytosis. *Traffic* 8 640–646 10.1111/j.1600-0854.2007.00568.x 17488288

[B41] Escobar V. D. Kuo Y. M. Orrison B. M. Giasson B. I. Nussbaum R. L. (2014). Transgenic mice expressing S129 phosphorylation mutations in alpha-synuclein. *Neurosci. Lett.* 563 96–100 10.1016/j.neulet.2014.01.033 24486885 PMC4059511

[B42] Febbraro F. Sahin G. Farran A. Soares S. Jensen P. H. Kirik D. (2013). Ser129D mutant alpha-synuclein induces earlier motor dysfunction while S129A results in distinctive pathology in a rat model of Parkinson’s disease. *Neurobiol. Dis.* 56 47–58 10.1016/j.nbd.2013.03.014 23567651

[B43] Ferrer I. Blanco R. Carmona M. Puig B. Barrachina M. Gomez C. (2001). Active, phosphorylation-dependent mitogen-activated protein kinase (MAPK/ERK), stress-activated protein kinase/c-Jun N-terminal kinase (SAPK/JNK), and p38 kinase expression in Parkinson’s disease and dementia with Lewy bodies. *J. Neural. Transm.* 108 1383–1396 10.1007/s007020100015 11810403

[B44] Fish K. J. Cegielska A. Getman M. E. Landes G. M. Virshup D. M. (1995). Isolation and characterization of human casein kinase I epsilon (CKI), a novel member of the CKI gene family. *J. Biol. Chem.* 270 14875–14883 10.1074/jbc.270.25.14875 7797465

[B45] Fitzgerald K. Bergeron M. Willits C. Bowers S. Aubele D. L. Goldbach E. (2013). Pharmacological inhibition of polo like kinase 2 (PLK2) does not cause chromosomal damage or result in the formation of micronuclei. *Toxicol. Appl. Pharmacol.* 269 1–7 10.1016/j.taap.2013.02.012 23466428

[B46] Fryer L. G. Parbu-Patel A. Carling D. (2002). The anti-diabetic drugs rosiglitazone and metformin stimulate AMP-activated protein kinase through distinct signaling pathways. *J. Biol. Chem.* 277 25226–25232 10.1074/jbc.M202489200 11994296

[B47] Fujiwara H. Hasegawa M. Dohmae N. Kawashima A. Masliah E. Goldberg M. S. (2002). alpha-Synuclein is phosphorylated in synucleinopathy lesions. *Nat. Cell Biol.* 4 160–164 10.1038/ncb748 11813001

[B48] Gandhi S. Muqit M. M. Stanyer L. Healy D. G. Abou-Sleiman P. M. Hargreaves I. (2006). PINK1 protein in normal human brain and Parkinson’s disease. *Brain* 129 1720–1731 10.1093/brain/awl114 16702191

[B49] Gao F. Chen D. Hu Q. Wang G. (2013). Rotenone directly induces BV2 cell activation via the p38 MAPK pathway. *PLoS ONE* 8:e72046 10.1371/journal.pone.0072046 PMC3748029 23977201

[B50] Gardet A. Benita Y. Li C. Sands B. E. Ballester I. Stevens C. (2010). LRRK2 is involved in the IFN-gamma response and host response to pathogens. *J. Immunol.* 185 5577–5585 10.4049/jimmunol.1000548 20921534 PMC3156100

[B51] Gasser T. (2009). Molecular pathogenesis of Parkinson disease: insights from genetic studies. *Expert Rev. Mol. Med.* 11:e22 10.1017/S1462399409001148 19631006

[B52] Giasson B. I. Covy J. P. Bonini N. M. Hurtig H. I. Farrer M. J. Trojanowski J. Q. (2006). Biochemical and pathological characterization of Lrrk2. *Ann. Neurol.* 59 315–322 10.1002/ana.20791 16437584

[B53] Glover M O’shaughnessy K. M. (2011). SPAK and WNK kinases: a new target for blood pressure treatment? *Curr. Opin. Nephrol. Hypertens*. 20 16–22 10.1097/MNH.0b013e32834132bc 21088576

[B54] Gomez-Santos C. Ferrer I. Reiriz J. Vinals F. Barrachina M. Ambrosio S. (2002). MPP^+^ increases alpha-synuclein expression and ERK/MAP-kinase phosphorylation in human neuroblastoma SH-SY5Y cells. *Brain Res.* 935 32–39 10.1016/S0006-8993(02)02422-8 12062470

[B55] Greene L. A. Levy O. Malagelada C. (2011). Akt as a victim, villain and potential hero in Parkinson’s disease pathophysiology and treatment. *Cell Mol. Neurobiol.* 31 969–978 10.1007/s10571-011-9671-9678 21547489 PMC3678379

[B56] Grenier K. Mclelland G. L. Fon E. A. (2013). Parkin- and PINK1-dependent mitophagy in neurons: will the real pathway please stand up? *Front. Neurol*. 4:100 10.3389/fneur.2013.00100 PMC3715719 23882257

[B57] Grunblatt E. Mandel S. Jacob-Hirsch J. Zeligson S. Amariglo N. Rechavi G. (2004). Gene expression profiling of parkinsonian substantia nigra pars compacta; alterations in ubiquitin-proteasome, heat shock protein, iron and oxidative stress regulated proteins, cell adhesion/cellular matrix and vesicle trafficking genes. *J. Neural. Transm.* 111 1543–1573 10.1007/s00702-004-0212-1 15455214

[B58] Guerreiro P. S. Huang Y. Gysbers A. Cheng D. Gai W. P. Outeiro T. F. (2013). LRRK2 interactions with alpha-synuclein in Parkinson’s disease brains and in cell models. *J. Mol. Med. (Berl.)* 91 513–522 10.1007/s00109-012-0984-y 23183827 PMC3611031

[B59] Gurevich E. V. Tesmer J. J. Mushegian A. Gurevich V. V. (2012). G protein-coupled receptor kinases: more than just kinases and not only for GPCRs. *Pharmacol. Ther.* 133 40–69 10.1016/j.pharmthera.2011.08.001 21903131 PMC3241883

[B60] Halliday G. Hely M. Reid W. Morris J. (2008). The progression of pathology in longitudinally followed patients with Parkinson’s disease. *Acta Neuropathol.* 115 409–415 10.1007/s00401-008-0344-8 18231798

[B61] Hassler R. (1938). Zur Pathologie der paralysis agitans und des post-enzephalitschen Parkinsonismus. *J. Psychol. Neurol.* 48 387–476

[B62] Healy D. G. Falchi M. O’sullivan S. S. Bonifati V. Durr A. Bressman S. (2008). Phenotype, genotype, and worldwide genetic penetrance of LRRK2-associated Parkinson’s disease: a case–control study. *Lancet Neurol.* 7 583–590 10.1016/S1474-4422(08)70117-0 18539534 PMC2832754

[B63] Hetman M. Gozdz A. (2004). Role of extracellular signal regulated kinases 1 and 2 in neuronal survival. *Eur. J. Biochem.* 271 2050–2055 10.1111/j.1432-1033.2004.04133.x 15153093

[B64] Higashi S. Biskup S. West A. B. Trinkaus D. Dawson V. L. Faull R. L. (2007). Localization of Parkinson’s disease-associated LRRK2 in normal and pathological human brain. *Brain Res.* 1155 208–219 10.1016/j.brainres.2007.04.034 17512502

[B65] Hongo H. Kihara T. Kume T. Izumi Y. Niidome T. Sugimoto H. (2012). Glycogen synthase kinase-3beta activation mediates rotenone-induced cytotoxicity with the involvement of microtubule destabilization. *Biochem. Biophys. Res. Commun.* 426 94–99 10.1016/j.bbrc.2012.08.042 22922102

[B66] Hoozemans J. J. Van Haastert E. S. Eikelenboom P. De Vos R. A. Rozemuller J. M. Scheper W. (2007). Activation of the unfolded protein response in Parkinson’s disease. *Biochem. Biophys. Res. Commun.* 354 707–711 10.1016/j.bbrc.2007.01.043 17254549

[B67] Hunot S. Vila M. Teismann P. Davis R. J. Hirsch E. C. Przedborski S. (2004). JNK-mediated induction of cyclooxygenase 2 is required for neurodegeneration in a mouse model of Parkinson’s disease. *Proc. Natl. Acad. Sci. U.S.A.* 101 665–670 10.1073/pnas.0307453101 14704277 PMC327205

[B68] Inglis K. J. Chereau D. Brigham E. F. Chiou S. S. Schobel S. Frigon N. L. (2009). Polo-like kinase 2 (PLK2) phosphorylates alpha-synuclein at serine 129 in central nervous system. *J. Biol. Chem.* 284 2598–2602 10.1074/jbc.C800206200 19004816 PMC2631975

[B69] Investigators., P. S. G. P. (2007). Mixed lineage kinase inhibitor CEP-1347 fails to delay disability in early Parkinson disease. *Neurology* 69 1480–1490 10.1212/01.wnl.0000277648.63931.c0 17881719

[B70] Ishii A. Nonaka T. Taniguchi S. Saito T. Arai T. Mann D. (2007). Casein kinase 2 is the major enzyme in brain that phosphorylates Ser129 of human alpha-synuclein: Implication for alpha-synucleinopathies. *FEBS Lett.* 581 4711–4717 10.1016/j.febslet.2007.08.067 17868672

[B71] Jaleel M. Nichols R. J. Deak M. Campbell D. G. Gillardon F. Knebel A. (2007). LRRK2 phosphorylates moesin at threonine-558: characterization of how Parkinson’s disease mutants affect kinase activity. *Biochem. J.* 405 307–317 10.1042/BJ20070209 17447891 PMC1904520

[B72] Kamal F. A. Travers J. G. Blaxall B. C. (2012). G protein-coupled receptor kinases in cardiovascular disease: why “where” matters. *Trends Cardiovasc. Med.* 22 213–219 10.1016/j.tcm.2012.07.023 23062971

[B73] Kanaoka Y. Kimura S. H. Okazaki I. Ikeda M. Nojima H. (1997). GAK: a cyclin G associated kinase contains a tensin/auxilin-like domain. *FEBS Lett.* 402 73–80 10.1016/S0014-5793(96)01484-6 9013862

[B74] Kawajiri S. Saiki S. Sato S. Hattori N. (2011). Genetic mutations and functions of PINK1. *Trends Pharmacol. Sci.* 32 573–580 10.1016/j.tips.2011.06.001 21784538

[B75] Kett L. R. Dauer W. T. (2012). Leucine-rich repeat kinase 2 for beginners: six key questions. *Cold Spring Harb. Perspect. Med.* 2 a009407 10.1101/cshperspect.a009407 PMC3282500 22393539

[B76] Kim J. Byun J. W. Choi I. Kim B. Jeong H. K. Jou I. (2013). PINK1 deficiency enhances inflammatory cytokine release from acutely prepared brain slices. *Exp. Neurobiol.* 22 38–44 10.5607/en.2013.22.1.38 23585721 PMC3620457

[B77] Knippschild U. Gocht A. Wolff S. Huber N. Lohler J. Stoter M. (2005). The casein kinase 1 family: participation in multiple cellular processes in eukaryotes. *Cell. Signal.* 17 675–689 10.1016/j.cellsig.2004.12.011 15722192

[B78] Kondapalli C. Kazlauskaite A. Zhang N. Woodroof H. I. Campbell D. G. Gourlay R. (2012). PINK1 is activated by mitochondrial membrane potential depolarization and stimulates Parkin E3 ligase activity by phosphorylating Serine 65. *Open Biol.* 2 120080 10.1098/rsob.120080. PMC3376738 22724072

[B79] Kozikowski A. P. Gaisina I. N. Petukhov P. A. Sridhar J. King L. T. Blond S. Y. (2006). Highly potent and specific GSK-3beta inhibitors that block tau phosphorylation and decrease alpha-synuclein protein expression in a cellular model of Parkinson’s disease. *Chem. Med. Chem.* 1 256–266 10.1002/cmdc.200500039 16892358

[B80] Kulich S. M. Chu C. T. (2001). Sustained extracellular signal-regulated kinase activation by 6-hydroxydopamine: implications for Parkinson’s disease. *J. Neurochem.* 77 1058–1066 10.1046/j.1471-4159.2001.00304.x 11359871 PMC1868550

[B81] Kyriakis J. M. Avruch J. (2012). Mammalian MAPK signal transduction pathways activated by stress and inflammation: a 10-year update. *Physiol. Rev.* 92 689–737 10.1152/physrev.00028.2011 22535895

[B82] Laplante M. Sabatini D. M. (2012). mTOR signaling in growth control and disease. *Cell* 149 274–293 10.1016/j.cell.2012.03.017 22500797 PMC3331679

[B83] Lee D. W. Zhao X. Yim Y. I. Eisenberg E. Greene L. E. (2008). Essential role of cyclin-G-associated kinase (Auxilin-2) in developing and mature mice. *Mol. Biol. Cell* 19 2766–2776 10.1091/mbc.E07-11-1115 18434600 PMC2441687

[B84] Lee H. J. Chung K. C. (2012). PINK1 positively regulates IL-1beta-mediated signaling through Tollip and IRAK1 modulation. *J. Neuroinflammation* 9 271 10.1186/1742-2094-9-271 PMC3533909 23244239

[B85] Lee K. W. Im J. Y. Woo J. M. Grosso H. Kim Y. S. Cristovao A. C. (2013). Neuroprotective and anti-inflammatory properties of a coffee component in the MPTP model of Parkinson’s disease. *Neurotherapeutics* 10 143–153 10.1007/s13311-012-0165-2 23296837 PMC3557367

[B86] Li X. Geng J. Liu J. (2014). Adiponectin offers protection against L166P mutant DJ-1-induced neuronal cytotoxicity mediated by APPL1-dependent AMPK activation. *Int. J. Neurosci.* 124 350–361 10.3109/00207454.2013.846340 24047115

[B87] Liao J. Wu C. X. Burlak C. Zhang S. Sahm H. Wang M. (2014). Parkinson disease-associated mutation R1441H in LRRK2 prolongs the “active state” of its GTPase domain. *Proc. Natl. Acad. Sci U.S.A.* . 10.1073/pnas.1323285111. PMC3964117 24591621

[B88] Lill C. M. Roehr J. T. Mcqueen M. B. Kavvoura F. K. Bagade S. Schjeide B. M. (2012). Comprehensive research synopsis and systematic meta-analyses in Parkinson’s disease genetics: the PDGene database. *PLoS Genet.* 8:e1002548 10.1371/journal.pgen.1002548 PMC3305333 22438815

[B89] Liu K. Shi N. Sun Y. Zhang T. Sun X. (2013). Therapeutic effects of rapamycin on MPTP-induced Parkinsonism in mice. *Neurochem. Res.* 38 201–207 10.1007/s11064-012-0909-8 23117422

[B90] Liu P. Wang X. Gao N. Zhu H. Dai X. Xu Y. (2010). G protein-coupled receptor kinase 5, overexpressed in the alpha-synuclein up-regulation model of Parkinson’s disease, regulates bcl-2 expression. *Brain Res.* 1307 134–141 10.1016/j.brainres.2009.10.036 19852948

[B91] Liu X. Cheng R. Verbitsky M. Kisselev S. Browne A. Mejia-Sanatana H. (2011). Genome-wide association study identifies candidate genes for Parkinson’s disease in an Ashkenazi Jewish population. *BMC Med. Genet.* 12:104 10.1186/1471-2350-12-104 PMC3166909 21812969

[B92] Lue L. F. Walker D. G. Adler C. H. Shill H. Tran H. Akiyama H. (2012). Biochemical increase in phosphorylated alpha-synuclein precedes histopathology of Lewy-type synucleinopathies. *Brain Pathol.* 22 745–756 10.1111/j.1750-3639.2012.00585.x 22369130 PMC4427521

[B93] Lynch-Day M. A. Mao K. Wang K. Zhao M. Klionsky D. J. (2012). The role of autophagy in Parkinson’s disease. *Cold Spring Harb. Perspect. Med.* 2 a009357 10.1101/cshperspect.a009357 PMC3312403 22474616

[B94] Malagelada C. Jin Z. H. Greene L. A. (2008). RTP801 is induced in Parkinson’s disease and mediates neuron death by inhibiting Akt phosphorylation/activation. *J. Neurosci.* 28 14363–14371 10.1523/JNEUROSCI.3928-08.2008 19118169 PMC3865436

[B95] Mandel S. A. Sagi Y. Amit T. (2007). Rasagiline promotes regeneration of substantia nigra dopaminergic neurons in post-MPTP-induced Parkinsonism via activation of tyrosine kinase receptor signaling pathway. *Neurochem. Res.* 32 1694–1699 10.1007/s11064-007-9351-8 17701352

[B96] Matsuda N. Sato S. Shiba K. Okatsu K. Saisho K. Gautier C. A. (2010). PINK1 stabilized by mitochondrial depolarization recruits Parkin to damaged mitochondria and activates latent Parkin for mitophagy. *J. Cell Biol.* 189 211–221 10.1083/jcb.200910140 20404107 PMC2856912

[B97] Mbefo M. K. Paleologou K. E. Boucharaba A. Oueslati A. Schell H. Fournier M. (2010). Phosphorylation of synucleins by members of the Polo-like kinase family. *J. Biol. Chem.* 285 2807–2822 10.1074/jbc.M109.081950 19889641 PMC2807335

[B98] McFarland N. R. Fan Z. Xu K. Schwarzschild M. A. Feany M. B. Hyman B. T. (2009). Alpha-synuclein S129 phosphorylation mutants do not alter nigrostriatal toxicity in a rat model of Parkinson disease. *J. Neuropathol. Exp. Neurol.* 68 515–524 10.1097/NEN.0b013e3181a24b53 19525899 PMC2753269

[B99] Meggio F. Pinna L. A. (2003). One-thousand-and-one substrates of protein kinase CK2? *FASEB J.* 17 349–368 10.1096/fj.02-0473rev 12631575

[B100] Melrose H. L. Kent C. B. Taylor J. P. Dachsel J. C. Hinkle K. M. Lincoln S. J. (2007). A comparative analysis of leucine-rich repeat kinase 2 (Lrrk2) expression in mouse brain and Lewy body disease. *Neuroscience* 147 1047–1058 10.1016/j.neuroscience.2007.05.027 17611037

[B101] Miklossy J. Arai T. Guo J. P. Klegeris A. Yu S. Mcgeer E. G. (2006). LRRK2 expression in normal and pathologic human brain and in human cell lines. *J. Neuropathol. Exp. Neurol.* 65 953–963 10.1097/01.jnen.0000235121.98052.54 17021400 PMC7185781

[B102] Moehle M. S. Webber P. J. Tse T. Sukar N. Standaert D. G. Desilva T. M. (2012). LRRK2 inhibition attenuates microglial inflammatory responses. *J. Neurosci.* 32 1602–1611 10.1523/JNEUROSCI.5601-11.2012 22302802 PMC3532034

[B103] Morales-Garcia J. A. Susin C. Alonso-Gil S. Perez D. I. Palomo V. Perez C. (2013). Glycogen synthase kinase-3 inhibitors as potent therapeutic agents for the treatment of Parkinson disease. *ACS Chem. Neurosci.* 4 350–360 10.1021/cn300182g 23421686 PMC3582296

[B104] Moreno J.A. Halliday M. Molloy C. Radford H. Verity N. Axten J.M. (2013). Oral treatment targeting the unfolded protein response prevents neurodegeneration and clinical disease in prion-infected mice. *Sci. Transl. Med*. 5 206ra138 10.1126/scitranslmed.3006767 24107777

[B105] Nagao M. Hayashi H. (2009). Glycogen synthase kinase-3beta is associated with Parkinson’s disease. *Neurosci. Lett.* 449 103–107 10.1016/j.neulet.2008.10.104 19007860

[B106] Nakamura S. Kawamoto Y. Nakano S. Akiguchi I. Kimura J. (1997). p35nck5a and cyclin-dependent kinase 5 colocalize in Lewy bodies of brains with Parkinson’s disease. *Acta Neuropathol.* 94 153–157 10.1007/s004010050687 9255390

[B107] Nalls M. A. Plagnol V. Hernandez D. G. Sharma M. Sheerin U. M. Saad M. (2011). Imputation of sequence variants for identification of genetic risks for Parkinson’s disease: a meta-analysis of genome-wide association studies. *Lancet* 377 641–649 10.1016/S0140-6736(10)62345-8. 21292315 PMC3696507

[B108] Narendra D. P. Jin S. M. Tanaka A. Suen D. F. Gautier C. A. Shen J. (2010). PINK1 is selectively stabilized on impaired mitochondria to activate Parkin. *PLoS Biol.* 8:e1000298 10.1371/journal.pbio.1000298 PMC2811155 20126261

[B109] Newhouse K. Hsuan S. L. Chang S. H. Cai B. Wang Y. Xia Z. (2004). Rotenone-induced apoptosis is mediated by p38 and JNK MAP kinases in human dopaminergic SH-SY5Y cells. *Toxicol. Sci.* 79 137–146 10.1093/toxsci/kfh089 14976342

[B110] Okochi M. Walter J. Koyama A. Nakajo S. Baba M. Iwatsubo T. (2000). Constitutive phosphorylation of the Parkinson’s disease associated alpha-synuclein. *J. Biol. Chem.* 275 390–397 10.1074/jbc.275.1.390 10617630

[B111] Olanow C. W. Brundin P. (2013). Parkinson’s disease and alpha synuclein: is Parkinson’s disease a prion-like disorder? *Mov. Disord* 28 31–40 10.1002/mds.25373 23390095

[B112] Oueslati A. Fournier M. Lashuel H. A. (2010). Role of post-translational modifications in modulating the structure, function and toxicity of alpha-synuclein: implications for Parkinson’s disease pathogenesis and therapies. *Prog. Brain Res.* 183 115–145 10.1016/S0079-6123(10)83007-9 20696318

[B113] Oueslati A. Schneider B. L. Aebischer P. Lashuel H. A. (2013). Polo-like kinase 2 regulates selective autophagic alpha-synuclein clearance and suppresses its toxicity in vivo. *Proc. Natl. Acad. Sci. U.S.A.* 110 E3945–E3954 10.1073/pnas.1309991110 23983262 PMC3799334

[B114] Paisan-Ruiz C. (2009). LRRK2 gene variation and its contribution to Parkinson disease. *Hum. Mutat.* 30 1153–1160 10.1002/humu.21038 19472409

[B115] Paisan-Ruiz C. Jain S. Evans E. W. Gilks W. P. Simon J. Van Der Brug M. (2004). Cloning of the gene containing mutations that cause PARK8-linked Parkinson’s disease. *Neuron* 44 595–600 10.1016/j.neuron.2004.10.023 15541308

[B116] Pan T. Rawal P. Wu Y. Xie W. Jankovic J. Le W. (2009). Rapamycin protects against rotenone-induced apoptosis through autophagy induction. *Neuroscience* 164 541–551 10.1016/j.neuroscience.2009.08.014 19682553

[B117] Pankratz N. Wilk J. B. Latourelle J. C. Destefano A. L. Halter C. Pugh E. W. (2009). Genomewide association study for susceptibility genes contributing to familial Parkinson disease. *Hum. Genet.* 124 593–605 10.1007/s00439-008-0582-9 18985386 PMC2627511

[B118] Perez D. I. Gil C. Martinez A. (2011). Protein kinases CK1 and CK2 as new targets for neurodegenerative diseases. *Med. Res. Rev*. 31 924–954 10.1002/med.20207 20577972

[B119] Persu A. Vikkula M. (2011). A genome-wide association study-derived candidate gene seeks replication: STK39. *J. Hypertens.* 29 434–436 10.1097/HJH.0b013e328344b6b3 21317723

[B120] Peters J. M. Mckay R. M. Mckay J. P. Graff J. M. (1999). Casein kinase I transduces Wnt signals. *Nature* 401 345–350 10.1038/43830 10517632

[B121] Pihlstrom L. Toft M. (2011). Genetic variability in SNCA and Parkinson’s disease. *Neurogenetics* 12 283–293 10.1007/s10048-011-0292-7 21800132

[B122] Polymeropoulos M. H. Lavedan C. Leroy E. Ide S. E. Dehejia A. Dutra A. (1997). Mutation in the alpha-synuclein gene identified in families with Parkinson’s disease. *Science* 276 2045–2047 10.1126/science.276.5321.2045 9197268

[B123] Pronin A. N. Morris A. J. Surguchov A. Benovic J. L. (2000). Synucleins are a novel class of substrates for G protein-coupled receptor kinases. *J. Biol. Chem.* 275 26515–26522 10.1074/jbc.M003542200 10852916

[B124] Qing H. Zhang Y. Deng Y. Mcgeer E. G. Mcgeer P. L. (2009). Lrrk2 interaction with alpha-synuclein in diffuse Lewy body disease. *Biochem. Biophys. Res. Commun.* 390 1229–1234 10.1016/j.bbrc.2009.10.126 19878656

[B125] Qu D. Rashidian J. Mount M. P. Aleyasin H. Parsanejad M. Lira A. (2007). Role of Cdk5-mediated phosphorylation of Prx2 in MPTP toxicity and Parkinson’s disease. *Neuron* 55 37–52 10.1016/j.neuron.2007.05.033 17610816

[B126] Recasens A. Dehay B. Bove J. Carballo-Carbajal I. Dovero S. Perez-Villalba A. (2014). Lewy body extracts from Parkinson disease brains trigger alpha-synuclein pathology and neurodegeneration in mice and monkeys. *Ann. Neurol.* 75 351–362 10.1002/ana.24066 24243558

[B127] Rhodes S. L. Sinsheimer J. S. Bordelon Y. Bronstein J. M. Ritz B. (2011). Replication of GWAS associations for GAK and MAPT in Parkinson’s disease. *Ann. Hum. Genet.* 75 195–200 10.1111/j.1469-1809.2010.00616.x 21058943 PMC3074465

[B128] Richardson C. Alessi D. R. (2008). The regulation of salt transport and blood pressure by the WNK-SPAK/OSR1 signalling pathway. *J. Cell Sci.* 121 3293–3304 10.1242/jcs.029223 18843116

[B129] Ries V. Henchcliffe C. Kareva T. Rzhetskaya M. Bland R. During M. J. (2006). Oncoprotein Akt/PKB induces trophic effects in murine models of Parkinson’s disease. *Proc. Natl. Acad. Sci. U.S.A.* 103 18757–18762 10.1073/pnas.0606401103 17116866 PMC1654135

[B130] Rodrigues E Silva A. M. Geldsetzer F. Holdorff B. Kielhorn F. W. Balzer-Geldsetzer M. Oertel W. H. (2010). Who was the man who discovered the “Lewy bodies”? *Mov. Disord*. 25 1765–1773 10.1002/mds.22956 20669275

[B131] Rozeboom A. M. Pak D. T. (2012). Identification and functional characterization of polo-like kinase 2 autoregulatory sites. *Neuroscience* 202 147–157 10.1016/j.neuroscience.2011.11.003 22100274 PMC3268898

[B132] Rudenko I. N. Chia R. Cookson M. R. (2012a). Is inhibition of kinase activity the only therapeutic strategy for LRRK2-associated Parkinson’s disease? *BMC Med.* 10:20 10.1186/1741-7015-10-20 PMC3308210 22361010

[B133] Rudenko I. N. Kaganovich A. Hauser D. N. Beylina A. Chia R. Ding J. (2012b). The G2385R variant of leucine-rich repeat kinase 2 associated with Parkinson’s disease is a partial loss-of-function mutation. *Biochem. J.* 446 99–111 10.1042/BJ20120637 22612223 PMC4667980

[B134] Ryu M. Y. Kim D. W. Arima K. Mouradian M. M. Kim S. U. Lee G. (2008). Localization of CKII beta subunits in Lewy bodies of Parkinson’s disease. *J. Neurol. Sci.* 266 9–12 10.1016/j.jns.2007.08.027 17884098

[B135] Sagi Y. Mandel S. Amit T. Youdim M. B. (2007). Activation of tyrosine kinase receptor signaling pathway by rasagiline facilitates neurorescue and restoration of nigrostriatal dopamine neurons in post-MPTP-induced parkinsonism. *Neurobiol. Dis.* 25 35–44 10.1016/j.nbd.2006.07.020 17055733

[B136] Sakamoto M. Arawaka S. Hara S. Sato H. Cui C. Machiya Y. (2009). Contribution of endogenous G-protein-coupled receptor kinases to Ser129 phosphorylation of alpha-synuclein in HEK293 cells. *Biochem. Biophys. Res. Commun.* 384 378–382 10.1016/j.bbrc.2009.04.130 19410557

[B137] Salvi M. Trashi E. Cozza G. Franchin C. Arrigoni G. Pinna L. A. (2012). Investigation on PLK2 and PLK3 substrate recognition. *Biochim. Biophys. Acta* 1824 1366–1373 10.1016/j.bbapap.2012.07.003 22828320

[B138] Samaranch L. Lorenzo-Betancor O. Arbelo J. M. Ferrer I. Lorenzo E. Irigoyen J. (2010). PINK1-linked parkinsonism is associated with Lewy body pathology. *Brain* 133 1128–1142 10.1093/brain/awq051 20356854

[B139] Saporito M. S. Brown E. M. Miller M. S. Carswell S. (1999). CEP-1347/KT-7515, an inhibitor of c-jun N-terminal kinase activation, attenuates the 1-methyl-4-phenyl tetrahydropyridine-mediated loss of nigrostriatal dopaminergic neurons in vivo. *J. Pharmacol*.* Exp. Ther.* 288 421–427 9918541

[B140] Saporito M. S. Thomas B. A. Scott R. W. (2000). MPTP activates c-Jun NH(2)-terminal kinase (JNK) and its upstream regulatory kinase MKK4 in nigrostriatal neurons in vivo. *J. Neurochem.* 75 1200–1208 10.1046/j.1471-4159.2000.0751200.x 10936203

[B141] Satake W. Nakabayashi Y. Mizuta I. Hirota Y. Ito C. Kubo M. (2009). Genome-wide association study identifies common variants at four loci as genetic risk factors for Parkinson’s disease. *Nat. Genet.* 41 1303–1307 10.1038/ng.485 19915576

[B142] Sato H. Kato T. Arawaka S. (2013). The role of Ser129 phosphorylation of alpha-synuclein in neurodegeneration of Parkinson’s disease: a review of in vivo models. *Rev. Neurosci.* 24 115–123 10.1515/revneuro-2012-0071 23314528

[B143] Schwab C. Demaggio A. J. Ghoshal N. Binder L. I. Kuret J. Mcgeer P. L. (2000). Casein kinase 1 delta is associated with pathological accumulation of tau in several neurodegenerative diseases. *Neurobiol. Aging* 21 503–510 10.1016/S0197-4580(00)00110-X 10924763

[B144] Seeburg D. P. Pak D. Sheng M. (2005). Polo-like kinases in the nervous system. *Oncogene* 24 292–298 10.1038/sj.onc.1208277 15640845

[B145] Sharma M. Ioannidis J. P. Aasly J. O. Annesi G. Brice A. Van Broeckhoven C. (2012). Large-scale replication and heterogeneity in Parkinson disease genetic loci. *Neurology* 79 659–667 10.1212/WNL.0b013e318264e353 22786590 PMC3414661

[B146] Sharma S. Bandopadhyay R. Lashley T. Renton A. E. Kingsbury A. E. Kumaran R. (2011). LRRK2 expression in idiopathic and G2019S positive Parkinson’s disease subjects: a morphological and quantitative study. *Neuropathol. Appl. Neurobiol.* 37 777–790 10.1111/j.1365-2990.2011.01187.x 21696411

[B147] Sheng Z. Zhang S. Bustos D. Kleinheinz T. Le Pichon C. E. Dominguez S. L. (2012). Ser1292 autophosphorylation is an indicator of LRRK2 kinase activity and contributes to the cellular effects of PD mutations. *Sci. Transl. Med.* 4 164ra161 10.1126/scitranslmed.3004485 23241745

[B148] Simon-Sanchez J. Schulte C. Bras J. M. Sharma M. Gibbs J. R. Berg D. (2009). Genome-wide association study reveals genetic risk underlying Parkinson’s disease. *Nat. Genet.* 41 1308–1312 10.1038/ng.487 19915575 PMC2787725

[B149] Smith P. D. Crocker S. J. Jackson-Lewis V. Jordan-Sciutto K. L. Hayley S. Mount M. P. (2003). Cyclin-dependent kinase 5 is a mediator of dopaminergic neuron loss in a mouse model of Parkinson’s disease. *Proc. Natl. Acad. Sci. U.S.A.* 100 13650–13655 10.1073/pnas.2232515100 14595022 PMC263868

[B150] Spencer B. Potkar R. Trejo M. Rockenstein E. Patrick C. Gindi R. (2009). Beclin 1 gene transfer activates autophagy and ameliorates the neurodegenerative pathology in alpha-synuclein models of Parkinson’s and Lewy body diseases. *J. Neurosci.* 29 13578–13588 10.1523/JNEUROSCI.4390-09.2009 19864570 PMC2812014

[B151] Spillantini M. G. Schmidt M. L. Lee V. M. Trojanowski J. Q. Jakes R. Goedert M. (1997). Alpha-synuclein in Lewy bodies. *Nature* 388 839–840 10.1038/42166 9278044

[B152] Tabara H. Naito Y. Ito A. Katsuma A. Sakurai M. A. Ohno S. (2011). Neonatal lethality in knockout mice expressing the kinase-dead form of the gefitinib target GAK is caused by pulmonary dysfunction. *PLoS ONE* 6:e26034 10.1371/journal.pone.0026034 PMC3192135 22022498

[B153] Takahashi M. Uchikado H. Caprotti D. Weidenheim K. M. Dickson D. W. Ksiezak-Reding H. (2006). Identification of G-protein coupled receptor kinase 2 in paired helical filaments and neurofibrillary tangles. *J. Neuropathol. Exp. Neurol.* 65 1157–1169 10.1097/01.jnen.0000248542.82681.12 17146290

[B154] Taymans J. M. (2012). The GTPase function of LRRK2. *Biochem. Soc. Trans.* 40 1063–1069 10.1042/BST20120133 22988866

[B155] Taymans J. M. Van Den Haute C. Baekelandt V. (2006). Distribution of PINK1 and LRRK2 in rat and mouse brain. *J. Neurochem.* 98 951–961 10.1111/j.1471-4159.2006.03919.x 16771836

[B156] Taymans J. M. Vancraenenbroeck R. Ollikainen P. Beilina A. Lobbestael E. De Maeyer M. (2011). LRRK2 kinase activity is dependent on LRRK2 GTP binding capacity but independent of LRRK2 GTP binding. *PLoS ONE* 6:e23207 10.1371/journal.pone.0023207 PMC3155532 21858031

[B157] Thomas T. Timmer M. Cesnulevicius K. Hitti E. Kotlyarov A. Gaestel M. (2008). MAPKAP kinase 2-deficiency prevents neurons from cell death by reducing neuroinflammation–relevance in a mouse model of Parkinson’s disease. *J. Neurochem.* 105 2039–2052 10.1111/j.1471-4159.2008.05310.x 18298661

[B158] Timmons S. Coakley M. F. Moloney A. M O’ Neill C. (2009). Akt signal transduction dysfunction in Parkinson’s disease. *Neurosci. Lett.* 467 30–35 10.1016/j.neulet.2009.09.055 19800394

[B159] Ugarte S. D. Lin E. Klann E. Zigmond M. J. Perez R. G. (2003). Effects of GDNF on 6-OHDA-induced death in a dopaminergic cell line: modulation by inhibitors of PI3 kinase and MEK. *J. Neurosci. Res.* 73 105–112 10.1002/jnr.10632 12815714

[B160] Ushiro H. Tsutsumi T. Suzuki K. Kayahara T. Nakano K. (1998). Molecular cloning and characterization of a novel Ste20-related protein kinase enriched in neurons and transporting epithelia. *Arch. Biochem. Biophys.* 355 233–240 10.1006/abbi.1998.0736 9675032

[B161] Valente E. M. Abou-Sleiman P. M. Caputo V. Muqit M. M. Harvey K. Gispert S. (2004). Hereditary early-onset Parkinson’s disease caused by mutations in PINK1. *Science* 304 1158–1160 10.1126/science.1096284 15087508

[B162] Walker D. G. Lue L. F. Adler C. H. Shill H. A. Caviness J. N. Sabbagh M. N. (2013). Changes in properties of serine 129 phosphorylated alpha-synuclein with progression of Lewy-type histopathology in human brains. *Exp. Neurol.* 240 190–204 10.1016/j.expneurol.2012.11.020 23201181 PMC3720241

[B163] Wang W. Shi L. Xie Y. Ma C. Li W. Su X. (2004). SP600125, a new JNK inhibitor, protects dopaminergic neurons in the MPTP model of Parkinson’s disease. *Neurosci. Res.* 48 195–202 10.1016/j.neures.2003.10.012 14741394

[B164] Wang W. Yang Y. Ying C. Li W. Ruan H. Zhu X. (2007). Inhibition of glycogen synthase kinase-3beta protects dopaminergic neurons from MPTP toxicity. *Neuropharmacology* 52 1678–1684 10.1016/j.neuropharm.2007.03.017 17517424

[B165] Wang Y. O’connell J. R. Mcardle P. F. Wade J. B. Dorff S. E. Shah S. J. (2009a). From the Cover: Whole-genome association study identifies STK39 as a hypertension susceptibility gene. *Proc. Natl. Acad. Sci. U.S.A.* 106 226–231 10.1073/pnas.0808358106 19114657 PMC2629209

[B166] Wang Y. Zhang Y. Wei Z. Li H. Zhou H. Zhang Z. (2009b). JNK inhibitor protects dopaminergic neurons by reducing COX-2 expression in the MPTP mouse model of subacute Parkinson’s disease. *J. Neurol. Sci.* 285 172–177 10.1016/j.jns.2009.06.034 19604516

[B167] Waxman E. A. Covy J. P. Bukh I. Li X. Dawson T. M. Giasson B. I. (2009). Leucine-rich repeat kinase 2 expression leads to aggresome formation that is not associated with alpha-synuclein inclusions. *J. Neuropathol. Exp. Neurol.* 68 785–796 10.1097/NEN.0b013e3181aaf4fd 19535993 PMC2722758

[B168] Waxman E. A. Giasson B. I. (2008). Specificity and regulation of casein kinase-mediated phosphorylation of alpha-synuclein. *J. Neuropathol. Exp. Neurol.* 67 402–416 10.1097/NEN.0b013e31816fc995 18451726 PMC2930078

[B169] Waxman E. A. Giasson B. I. (2011). Characterization of kinases involved in the phosphorylation of aggregated alpha-synuclein. *J. Neurosci. Res.* 89 231–247 10.1002/jnr.22537 21162130 PMC4484797

[B170] West A. B. Cowell R. M. Daher J. P. Moehle M. S. Hinkle K. M. Melrose H. L. (2014). Differential LRRK2 expression in the cortex, striatum, and substantia nigra in transgenic and nontransgenic rodents. *J. Comp. Neurol*. 522 Spc1 10.1002/cne.23583 PMC4076169 24633735

[B171] West A. B. Moore D. J. Biskup S. Bugayenko A. Smith W. W. Ross C. A. (2005). Parkinson’s disease-associated mutations in leucine-rich repeat kinase 2 augment kinase activity. *Proc. Natl. Acad. Sci. U.S.A.* 102 16842–16847 10.1073/pnas.0507360102 16269541 PMC1283829

[B172] Wills J. Jones J. Haggerty T. Duka V. Joyce J. N. Sidhu A. (2010). Elevated tauopathy and alpha-synuclein pathology in postmortem Parkinson’s disease brains with and without dementia. *Exp. Neurol.* 225 210–218 10.1016/j.expneurol.2010.06.017 20599975 PMC2922478

[B173] Winkles J. A. Alberts G. F. (2005). Differential regulation of polo-like kinase 1, 2, 3, and 4 gene expression in mammalian cells and tissues. *Oncogene* 24 260–266 10.1038/sj.onc.1208219 15640841

[B174] Woodroof H. I. Pogson J. H. Begley M. Cantley L. C. Deak M. Campbell D. G. (2011). Discovery of catalytically active orthologues of the Parkinson’s disease kinase PINK1: analysis of substrate specificity and impact of mutations. *Open Biol.* 1 110012 10.1098/rsob.110012 PMC3352081 22645651

[B175] Wu Y. Li X. Zhu J. X. Xie W. Le W. Fan Z. (2011). Resveratrol-activated AMPK/SIRT1/autophagy in cellular models of Parkinson’s disease. *Neurosignals* 19 163–174 10.1159/000328516 21778691 PMC3699815

[B176] Xing B. Liu M. Bing G. (2007). Neuroprotection with pioglitazone against LPS insult on dopaminergic neurons may be associated with its inhibition of NF-kappaB and JNK activation and suppression of COX-2 activity. *J. Neuroimmunol.* 192 89–98 10.1016/j.jneuroim.2007.09.029 17976742

[B177] Xiong N. Jia M. Chen C. Xiong J. Zhang Z. Huang J. (2011). Potential autophagy enhancers attenuate rotenone-induced toxicity in SH-SY5Y. *Neuroscience* 199 292–302 10.1016/j.neuroscience.2011.10.031 22056603

[B178] Yan Y. Nguyen H. Dalmasso G. Sitaraman S. V. Merlin D. (2007). Cloning and characterization of a new intestinal inflammation-associated colonic epithelial Ste20-related protein kinase isoform. *Biochim. Biophys. Acta* 1769 106–116 10.1016/j.bbaexp.2007.01.003 17321610 PMC1865517

[B179] Yuskaitis C. J. Jope R. S. (2009). Glycogen synthase kinase-3 regulates microglial migration, inflammation, and inflammation-induced neurotoxicity. *Cell. Signal.* 21 264–273 10.1016/j.cellsig.2008.10.014 19007880 PMC2630396

[B180] Zhai A. Zhu X. Wang X. Chen R. Wang H. (2013). Secalonic acid A protects dopaminergic neurons from 1-methyl-4-phenylpyridinium (MPP(+))-induced cell death via the mitochondrial apoptotic pathway. *Eur. J. Pharmacol.* 713 58–67 10.1016/j.ejphar.2013.04.029 23665112

[B181] Zhou J. Broe M. Huang Y. Anderson J. P. Gai W. P. Milward E. A. (2011). Changes in the solubility and phosphorylation of alpha-synuclein over the course of Parkinson’s disease. *Acta Neuropathol.* 121 695–704 10.1007/s00401-011-0815-1 21400129

[B182] Zhu J. H. Guo F. Shelburne J. Watkins S. Chu C. T. (2003). Localization of phosphorylated ERK/MAP kinases to mitochondria and autophagosomes in Lewy body diseases. *Brain Pathol.* 13 473–481 10.1111/j.1750-3639.2003.tb00478.x 14655753 PMC1911206

[B183] Zhu J. H. Kulich S. M. Oury T. D. Chu C. T. (2002). Cytoplasmic aggregates of phosphorylated extracellular signal-regulated protein kinases in Lewy body diseases. *Am. J. Pathol.* 161 2087–2098 10.1016/S0002-9440(10)64487-2 12466125 PMC1850911

[B184] Zimprich A. Biskup S. Leitner P. Lichtner P. Farrer M. Lincoln S. (2004). Mutations in LRRK2 cause autosomal-dominant parkinsonism with pleomorphic pathology. *Neuron* 44 601–607 10.1016/j.neuron.2004.11.005 15541309

